# On-surface cyclodehydrogenation reaction pathway determined by selective molecular deuterations†

**DOI:** 10.1039/d1sc04908a

**Published:** 2021-11-16

**Authors:** Chuanxu Ma, Zhongcan Xiao, Peter V. Bonnesen, Liangbo Liang, Alexander A. Puretzky, Jingsong Huang, Marek Kolmer, Bobby G. Sumpter, Wenchang Lu, Kunlun Hong, Jerzy Bernholc, An-Ping Li

**Affiliations:** Center for Nanophase Materials Sciences, Oak Ridge National Laboratory Oak Ridge TN 37831 USA huangj3@ornl.gov apli@ornl.gov; Hefei National Laboratory for Physical Sciences at the Microscale, Synergetic Innovation Center of Quantum Information & Quantum Physics, University of Science and Technology of China Hefei Anhui 230026 China; Department of Physics, North Carolina State University Raleigh NC 27695 USA; Ames Laboratory, U.S. Department of Energy Ames IA 50011 USA; Computational Sciences and Engineering Division, Oak Ridge National Laboratory Oak Ridge TN 37831 USA

## Abstract

Understanding the reaction mechanisms of dehydrogenative C_aryl_–C_aryl_ coupling is the key to directed formation of π-extended polycyclic aromatic hydrocarbons. Here we utilize isotopic labeling to identify the exact pathway of cyclodehydrogenation reaction in the on-surface synthesis of model atomically precise graphene nanoribbons (GNRs). Using selectively deuterated molecular precursors, we grow seven-atom-wide armchair GNRs on a Au(111) surface that display a specific hydrogen/deuterium (H/D) pattern with characteristic Raman modes. A distinct hydrogen shift across the fjord of C_aryl_–C_aryl_ coupling is revealed by monitoring the ratios of gas-phase by-products of H_2_, HD, and D_2_ with *in situ* mass spectrometry. The identified reaction pathway consists of a conrotatory electrocyclization and a distinct [1,9]-sigmatropic D shift followed by H/D eliminations, which is further substantiated by nudged elastic band simulations. Our results not only clarify the cyclodehydrogenation process in GNR synthesis but also present a rational strategy for designing on-surface reactions towards nanographene structures with precise hydrogen/deuterium isotope labeling patterns.

## Abstract 2

Selective deuterations were exploited to synthesize graphene nanoribbons on Au(111) surface with a specific H/D pattern on edges, allowing the determination of cyclodehydrogenation reaction pathway within the framework of pericyclic reactions.

## Graphical abstract

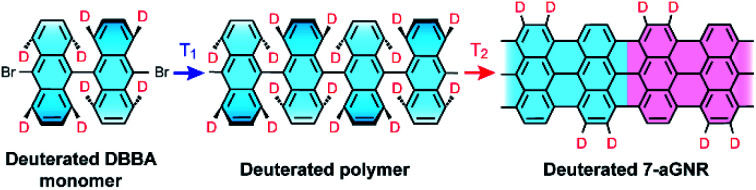

## Introduction

Intermolecular dehydrogenative C_aryl_–C_aryl_ couplings between (hetero)arenes and particularly the associated intramolecular annulative reactions have become one of the most powerful approaches in modern organic synthetic chemistry to assemble diverse precursor molecules into π-extended polycyclic aromatic hydrocarbons (PAHs) or nanographenes.^1,2^ Despite over a century's extensive experimental studies augmented by modern theoretical calculations, a full understanding of the underlying mechanisms of various dehydrogenative coupling reactions, such as the oxidative aromatic couplings^3,4^ and the Scholl reactions,^5–7^ still remains elusive due in large part to the complex and often uncontrollable environment in solution-phase reactions.^8^ Clarification of the exact mechanisms for dehydrogenative coupling reactions stands as a critical need to realize π-extended graphitic scaffolds with designed structures and desirable functions.^1^

Conventional solution-phase dehydrogenative C_aryl_–C_aryl_ couplings have recently been expanded to on-surface thermally-triggered cyclodehydrogenation reactions,^1,9,10^ which allow precise assembly of rationally designed molecular precursors into a gamut of well-defined sp^2^-bonded graphitic nanoarchitectures, such as fullerenes,^11,12^ nanographenes,^13,14^ carbon nanotubes,^15^ and graphene nanoribbons (GNRs).^16–25^ In the latter case, the reported two-step approach^16^ opens a new era for the synthesis of atomically precise GNRs of various topologies and sizes on noble metal surfaces.^20–24,26–28^ A prominent case is the seven-carbon-wide armchair GNR (7-aGNR) made from 10,10′-dibromo-9,9′-bianthracene (DBBA) molecular precursors on a Au(111) surface through dehalogenation/polymerization^29^ followed by cyclodehydrogenation.^16,30–33^ The cyclodehydrogenation step presents extremely high regioselectivity resulting from the steric hindrance effect between neighboring anthrylene units, making it a prototypical reaction for unraveling the mechanisms by experimental studies along with density functional theory (DFT) calculations. In 2011, Björk *et al.* proposed theoretically a reaction pathway where new C_aryl_–C_aryl_ bond formation is followed by simultaneous elimination of two H atoms from the two bonding C atoms to the Au(111) substrate.^34^ Using Ag(111) to model the cyclodehydrogenation on Au(111) surface, Blankenburg *et al.* put forth in 2012 a different pathway combining in-tandem H eliminations and a [1,2] nearest neighbor H shift after the C_aryl_–C_aryl_ bond formation.^35^ Recently, our study suggested yet another pathway consisting of H eliminations after a [1,3]-sigmatropic H shift following a scanning tunneling microscope (STM) tip directed ring closure for quasi-freestanding polymers.^36^ Despite last decade's advances, a consensus is still lacking on the exact reaction mechanisms for the on-surface cyclodehydrogenation.

Inspired by the broad applications of selective deuteration, ^1^H → ^2^H(D), in the fields of materials science, biology, and chemistry,^37–39^ we devise in this work a viable strategy based on rationally deuterated precursors allowing us to pinpoint the cyclodehydrogenation pathway during the synthesis of 7-aGNRs on Au(111) surface. Using Raman spectroscopy and DFT calculations, we identify the specific H/D pattern in the fabricated 7-aGNRs through its characteristic breathing-like Raman modes. Furthermore, based on systematic *in situ* mass spectrometry measurements of the ratios between gas-phase cyclodehydrogenation by-products and climbing image nudged elastic band (CI-NEB) simulations of the energy profiles in different reaction pathways, we determine that the exact reaction pathway consists of a conrotatory electrocyclization and a distinct [1,9]-sigmatropic D shift followed by subsequent H/D eliminations. The combined experimental and theoretical evidence elucidates the underlying cyclodehydrogenation pathway for the first time within the framework of pericyclic reactions.

## Results

**Fig. 1:**
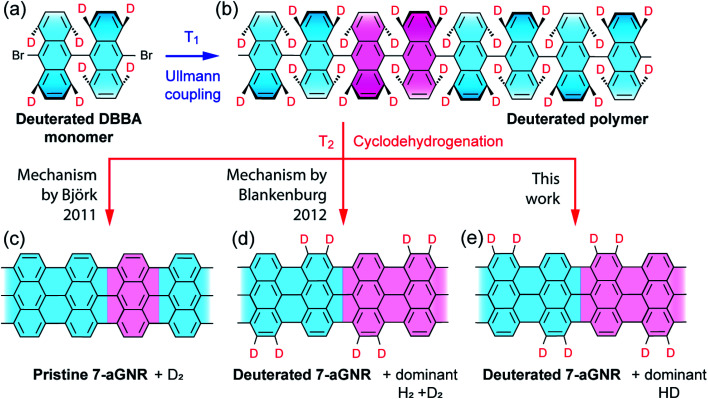
Schematic illustration of proposed reaction pathways during synthesis of 7-aGNRs. (a) Deuterated 10,10′-dibromo-9,9′-bianthracene-1,1′,4,4′,5,5′,8,8′-*d*_8_ (*d*_8_-DBBA) molecular monomer at selected positions used to differentiate between three possible mechanisms (see details below). (b) Deuterated polyanthrylene polymer obtained after Ullmann coupling at *T*_1_ = 470 K. (c)–(e) Pristine and deuterated 7-aGNRs obtained after cyclodehydrogenation at *T*_2_ = 670 K following different hydrogen elimination and/or shift mechanisms. In (a) and (b), tilting anthrylene units are indicated by different bond thicknesses and color gradient. In (b) through (e), unit cells in polyanthrylene and deuterated 7-aGNRs are highlighted in pink. Only the D atoms are highlighted while the H atoms are omitted for clarity.

We start by showing a holistic view of the possible on-surface reaction pathways that convert the selectively deuterated molecule precursors to 7-aGNRs. As illustrated in Fig. 1, the 10,10′-dibromo-9,9′-bianthracene-1,1′,4,4′,5,5′,8,8′-*d*_8_ (*d*_8_-DBBA) monomers (Fig. 1a) are converted to deuterated polyanthrylene in the Ullmann polymerization at *T*_1_ = 470 K (Fig. 1b), and then to 7-aGNRs in the cyclodehydrogenation at *T*_2_ = 670 K (Fig. 1c–e).^16,34–36^ Depending on the cyclodehydrogenation pathways, use of selectively deuterated precursors would result in three possible outcomes with specific patterns of edge passivation and different gas-phase by-products. First, if the cyclodehydrogenation proceeds with a pathway of simultaneous elimination of two H atoms as proposed by Björk *et al.*^34^ (Fig. S1†), the 7-aGNRs would be pristine without any D atoms as show in Fig. 1c. Second, if the reaction pathway follows the in-tandem H eliminations and a [1,2] nearest neighbor H shift as proposed by Blankenburg *et al.*^35^ (Fig. S1†), a deuterated ribbon structure would be produced with a specific H/D pattern at the edges as shown in Fig. 1d. Third, with a new pathway to be revealed below, the 7-aGNRs would have a H/D pattern of Fig. 1e equivalent to Fig. 1d but with distinctly different gas-phase by-products. Below we differentiate these cyclodehydrogenation pathways using Raman and *in situ* mass spectrometry measurements along with theoretical calculations.

**Fig. 2:**
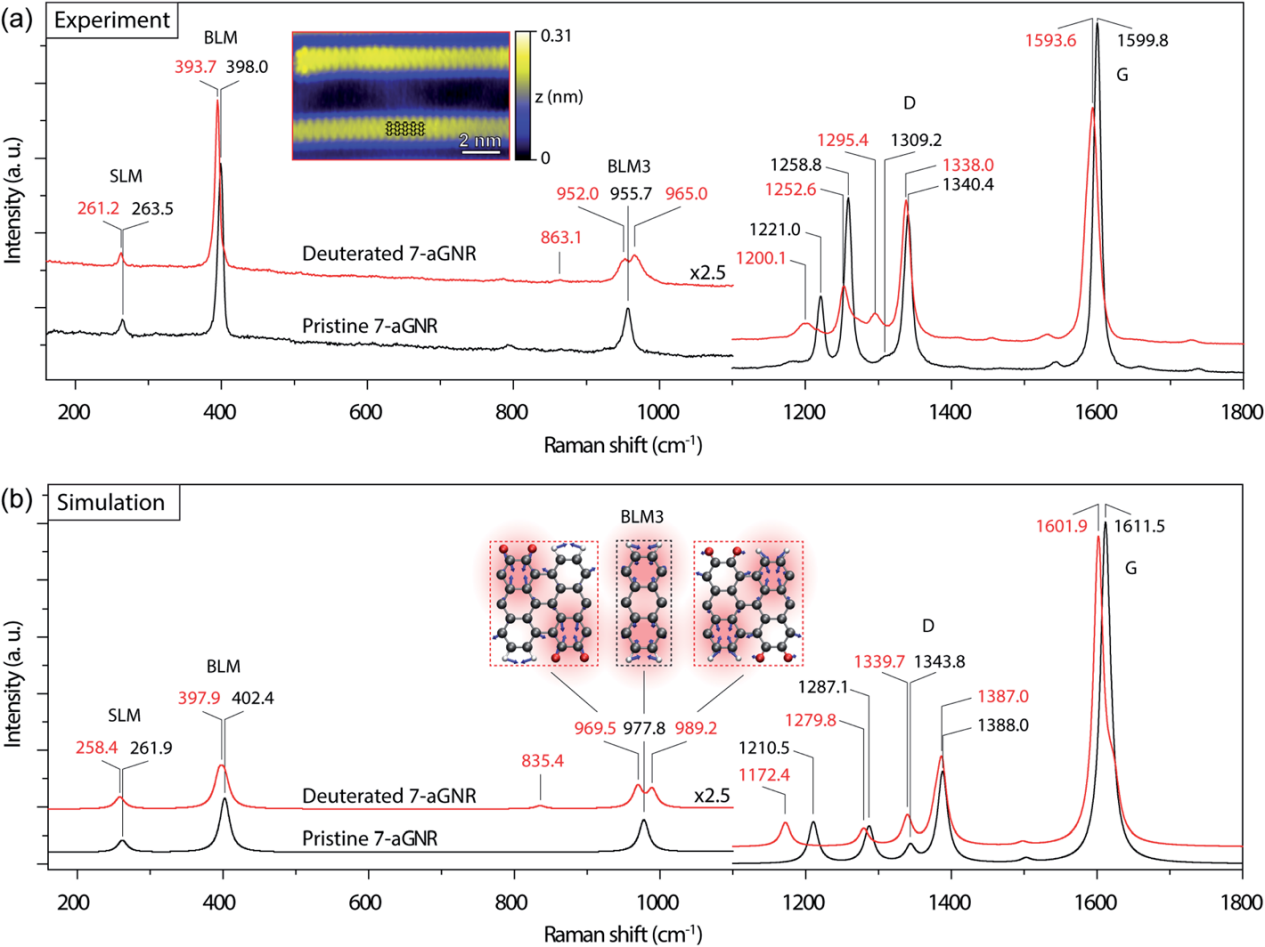
Raman spectra of 7-aGNRs. (a) Experimental Raman spectra (532 nm laser excitation, ∼0.7 cm^−1^ spectral resolution) of densely packed pristine (black) and deuterated (red) 7-aGNR samples. The main peak positions in the two spectra, corresponding to the vibrational modes of SLM, BLM, BLM3, D-like and G-like bands, are labeled in black and red, respectively. Inset: high-resolution STM image of individual deuterated 7-aGNRs (*V*_s_ = −0.3 V, *I*_t_ = 70 pA), with the structural model superimposed. (b) Simulated Raman spectra of pristine (black) and deuterated (red) 7-aGNRs with Lorentzian broadening of 16 cm^−1^. The main peaks in the two spectra are marked in black and red, respectively. The peak splitting in BLM3 is clearly noticeable. Inset: visualization by displacement vectors for the vibrational modes of BLM3 in the pristine and deuterated 7-aGNRs (grey for C, red for D, and white for H).

The *d*_8_-DBBA monomer we employed for 7-aGNR fabrication was synthesized by adopting a superacid-catalyzed H/D exchange reaction.^40^ Due to differences in the H/D exchange rates for the different carbon atoms of the anthracene moiety, the 1,4,5,8-positions are nearly selectively deuterated relative to the 2,3,6,7-positions (see Fig. S2–S5† for position indices). By nuclear magnetic resonance (NMR) analysis, the deuteration level was found to be nearly complete at the 1,8-positions (*ca.* 91 atom% D) and the 4,5-positions (*ca.* 97 atom% D), but significantly lower at the 2,7-positions (<1 atom% D) and the 3,6-positions (*ca.* 10 atom% D) (Fig. S2–S5†). Despite these high isotopic purities, we raise a caveat upfront that our isotope-dependent experimental results to be presented below may be slightly affected by the lack of 100% perfectly pure H and D at 2,3,6,7- and 1,4,5,8-positions, respectively. The fabricated 7-aGNRs were characterized by STM on Au(111) surface. The STM images show a high quality of 7-aGNRs (Fig. 2a inset and Fig. S6†), with very similar atomic and electronic structures to those of the pristine 7-aGNRs,^16,32,33,36,41^ and thus the analysis of our STM data here cannot determine whether the 7-aGNRs are deuterated or pristine.

We then examined Raman spectroscopy of these 7-aGNRs, because of its chemical specificity and sensitivity to possible mass changes from deuteration on the edges.^42,43^Fig. 2a compares the Raman spectra acquired from densely packed 7-aGNRs synthesized from pristine DBBA and *d*_8_-DBBA molecules, respectively (for higher frequency region, see Fig. S7†). Some differences can be clearly seen. First, the deuterated 7-aGNRs show noticeable Raman redshifts as compared to the pristine counterparts. For example, the shear-like mode (SLM) is shifted from 263.5 to 261.2 cm^−1^, while the breathing-like mode (BLM) is shifted from 398.0 to 393.7 cm^−1^. The peaks at the D- and G-like bands also show similar redshifts. The redshifts are the direct evidence of mass change due to the presence of D atoms on the ribbons. Second, more striking changes occur at the three-nodal BLM, namely, the BLM3. The BLM3 peak of the pristine 7-aGNRs is located at 955.7 cm^−1^,^41^ whereas in the deuterated GNRs it splits into two peaks, at 952.0 and 965.0 cm^−1^. The peak splitting of BLM3 suggests symmetry breaking due to the H/D-patterned 7-aGNR edges, further confirming the presence of D atoms.

Due to the dehydrogenative C_aryl_–C_aryl_ coupling, the D atoms can only exist on the ribbon edges. We considered four H/D patterns, including the one given in Fig. 1d and e along with three other possibilities (Fig. S8†). To determine the exact H/D pattern for the obtained 7-aGNRs, we performed DFT simulations of Raman spectra for all four H/D patterns together with the pristine 7-aGNRs. While all four H/D patterns give rise to the expected redshifts (Fig. S8†), only the pattern shown in Fig. 1d,e and 2b inset produces the splitting of BLM3. As shown in Fig. 2b, the simulated BLM3 consists of two peaks respectively at 969.5 and 989.2 cm^−1^, giving a relatively large splitting of 19.7 cm^−1^ that can be observed despite Lorentzian broadening. The corresponding vibrational modes, illustrated as insets in Fig. 2b, clearly show the symmetry breaking in the H/D-patterned 7-aGNRs. In comparison, the simulated BLM shows a much smaller splitting of 7.7 cm^−1^ (Fig. S8†), which collapses into one peak at 397.9 cm^−1^ after Lorentzian broadening. The experimentally measured BLM peak of deuterated GNRs is indeed slightly broader than that of the pristine counterpart (Fig. S9†). Based on this analysis, we can rule out the mechanism of simultaneous elimination of two hydrogen atoms from the bonding C positions^34^ since its putative loss of all D atoms during cyclodehydrogenation (Fig. S1†) does not agree with our Raman results.

**Fig. 3:**
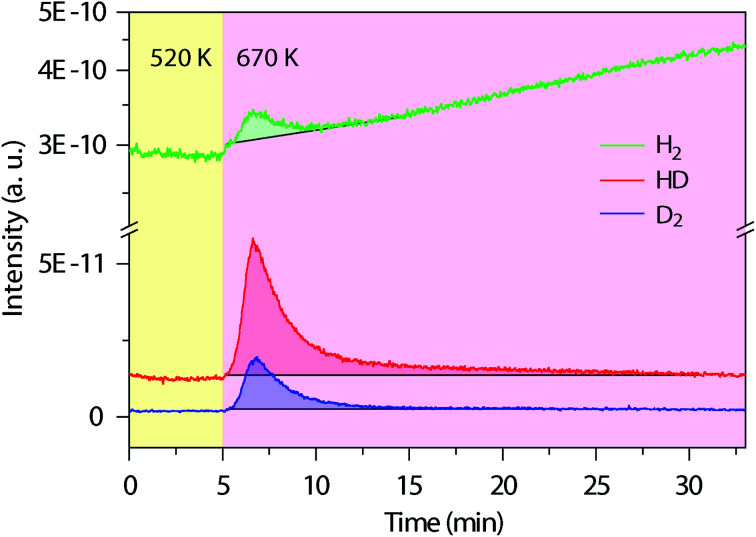
*In situ* mass spectra evolution with time during thermal annealing. Simultaneously measured mass spectra for the gas-phase by-products of H_2_ (2 amu), HD (3 amu) and D_2_ (4 amu) during cyclodehydrogenation of the deuterated polyanthrylene. The temperature changes from 520 to 670 K to trigger cyclodehydrogenation, first pre-annealing the polymers at 520 K for 1 h to remove the residual bromine (Br) on the Au(111) surface (Fig. S10†). The shadowed parts mark the peak areas, with the black lines from the bare Au(111) substrate as background (Fig. S11†), giving the average ratios of H_2_/HD/D_2_ = 1.25 : 2.70 : 1.

Next, we used *in situ* mass spectrometry to examine the gas-phase by-products during the cyclodehydrogenation process. Fig. 3 shows representative mass spectra of 2, 3 and 4 atomic mass units (amu), corresponding to H_2_, HD, and D_2_, respectively, measured with time during the cyclodehydrogenation process of the deuterated polyanthrylene (results for the pristine counterpart and clean Au(111) substrate are shown in Fig. S10 and S11†, respectively). Consistent H_2_/HD/D_2_ ratios of 1.25 : 2.70 : 1 can be obtained at varying GNR coverages (Fig. S12†). Different reaction pathways can lead to distinct gas-phase by-products (Fig. S13†). For example, the direct elimination pathway (Björk 2011, ref. 34) would form D_2_ only (Fig. S1†), while in the [1,2] hydrogen shift pathway (Blankenburg 2012, ref. 35), two immediately neighboring H atoms on the edges eliminate to give prevailing H_2_, leaving the well-separated D atoms to form D_2_ (Fig. S1†). However, the mass spectra show that HD is dominant, obviously different from the components expected from the direct elimination pathway (D_2_) and the [1,2] hydrogen shift pathway (H_2_ and D_2_). In the ultrahigh vacuum (UHV) of less than 1 × 10^−10^ torr, the Au(111) surface does not facilitate scrambling between reabsorbed and dissociated H_2_ and D_2_ (Fig. S14† and ref. 44). Thus, the observed HD signal is formed directly from the combinatively eliminated H and D atoms during the cyclodehydrogenation process. These reaction conditions are different from the recently reported H/D isotope exchange for PAHs on Au(111) in the presence of D_2_ gas of various partial pressures (10^−5^ to 10^−1^ torr).^45^ The D_2_ molecules present in the gas phase can shift the physisorption toward chemisorption giving dissociated D atoms on the Au(111) surface to facilitate the H/D isotope exchange (Fig. S14†). Therefore, the experimentally observed dominant HD signal indicates a distinct reaction pathway involving a hydrogen shift across the fjord from the newly formed bond to the edge of a neighboring anthrylene unit (Fig. S15†).

**Fig. 4:**
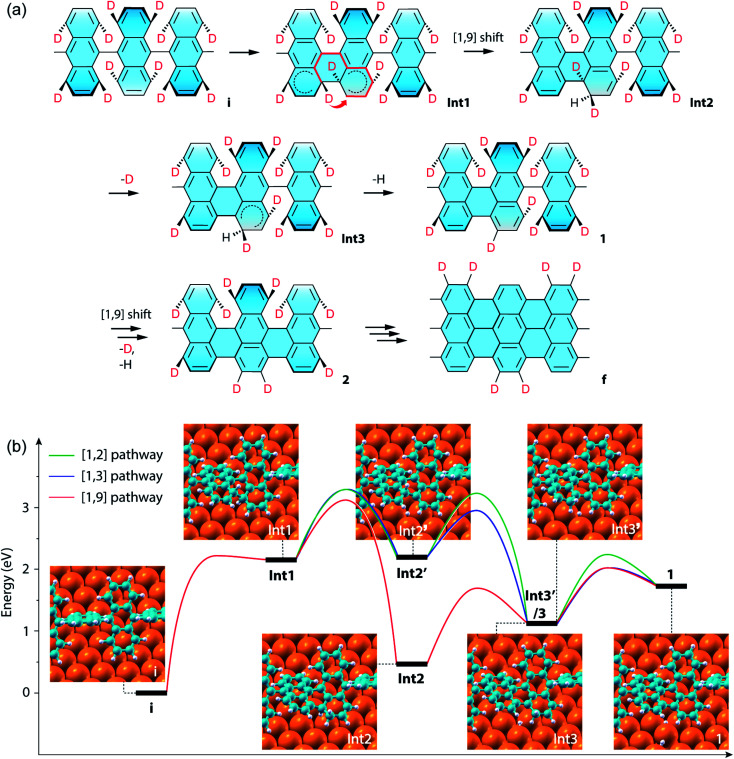
CI-NEB simulations of the cyclodehydrogenation process in the formation of the deuterated 7-aGNR. (a) Proposed reaction pathway of a conrotatory electrocyclization and a [1,9]-sigmatropic hydrogen shift. In the structure for Int1, the short thick arrow indicates the sigmatropic hydrogen shift across the fjord of bond formation, and the thick red lines show the shortest path of conjugation for the sigmatropic hydrogen shift. (b) Energy profiles of the exemplar first C_aryl_–C_aryl_ bond formation by three different pathways, namely the [1,2], [1,3] and [1,9] pathways (Fig. S15†), which respectively include [1,2], [1,3]-sigmatropic, and [1,9]-sigmatropic hydrogen shifts. The corresponding structural models of the intermediate states are also shown as insets, with the periodic direction along the [110] direction of the Au surface (Fig. S16†). The three pathways share the same initial (state i) and final (state 1) states, while the [1,2] and [1,3] pathways have the same Int1 and Int2′, and the [1,3] and [1,9] pathways have the same Int1 and Int3. Note that the energy difference between Int3′ for the [1,2] pathway and Int3 is only 0.01 eV.

The above results of Raman spectroscopy, mass-spectrometry, and the ensuing CI-NEB simulations (for technical details, see the ESI†) point to a new cyclodehydrogenation pathway as shown in Fig. 4a. The neighboring anthrylene units in the initial state i first rotate about the polymer axis to approach each other. Immediately following that, they undergo a conrotatory electrocyclization by involving a total number of 4*n* π-electrons in the two neighboring anthrylene units,^46^ allowing two benzyne groups (C_6_H_2_D_2_) on the same side to form a single C_sp^3^_–C_sp^3^_ bond, giving Int1. Then the D atom on the top side of the newly formed bond migrates by a [1,9]-sigmatropic hydrogen shift across the fjord to an edge C atom of a neighboring anthrylene unit, giving Int2. Next, the D atom at the other bonding C and the H atom at the edge C, both facing down to the Au substrate, are sequentially eliminated as ad-atoms on the Au surface, respectively giving Int3 and state 1, thereby restoring the aromaticity of the scaffold. Subsequently, a neighboring C_aryl_–C_aryl_ bond forms in the same way as in a one-side domino-like mechanism,^32,35,47^ giving state 2 with two D atoms located at the previously down-tilted anthrylene edge. Finally, the other side of the polymer goes through the same reaction path, which leads to state f with the H/D pattern as identified by Raman spectroscopy and DFT calculations. If two eliminated hydrogen atoms are closely separated, they are located in the potential well of interatomic attraction,^48^ with magnitude of force decreasing with increasing interatomic separations. In our proposed pathway, the sequentially eliminated H and D atoms are one C–C bond apart, indicating they prefer to form HD by proximity coupling. Therefore, the proposed hydrogen [1,9]-sigmatropic shift pathway is entirely consistent with the specific H/D pattern at the edges of 7-aGNRs identified by Raman and the dominant HD in the gas-phase by-products detected by mass spectrometry.

We further compared in Fig. 4b the CI-NEB energy profiles for the cyclodehydrogenation pathways that involve the [1,2] hydrogen shift (*i.e.*, [1,2] pathway) as proposed by Blankenburg *et al.*,^35^ the new [1,3]-sigmatropic hydrogen shift (*i.e.*, [1,3] pathway), and the most favorable [1,9]-sigmatropic hydrogen shift (*i.e.*, [1,9] pathway), respectively (Table S1 and Fig. S15†). We only consider the suprafacial and not the antarafacial sigmatropic hydrogen shifts since the latter scenario can be excluded based on the gas-phase by-products and steric hindrances (Fig. S1 and S13†). The three pathways share the same initial (state i) and final (state 1) states and thus can lead to the same H/D pattern identified by the Raman spectroscopy. However, the three reactions proceed with different elementary steps. Unlike the [1,9] pathway, in the [1,2] and [1,3] pathways, Int1 is first converted to Int2′ by eliminating the hydrogen atom facing down to the Au(111) surface before hydrogen shifts. Int2′ is then converted to Int3′ if the hydrogen atom facing up at the other bonding C undergoes a [1,2] nearest neighbor shift to the edge of the same anthrylene unit, or to Int3 if the same atom undergoes a [1,3]-sigmatropic shift across the fjord to the edge of a neighboring anthrylene unit. The difference between the [1,3]- and [1,9]-sigmatropic shifts lies in the order of elimination of the bottom-side hydrogen atom to the Au substrate. In the case of the [1,3] pathway (Fig. S1 and S15†), the elimination of the bottom-side hydrogen atom allows the shortest path of conjugation for the top-side hydrogen shift to be established along only three C atoms. As can be seen from Fig. 4b, the three pathways all share the same first step of C_sp^3^_–C_sp^3^_ bond formation (*i.e.*, state i to Int1), which has an energy barrier of 2.23 eV. The subsequent hydrogen elimination and shift steps, regardless of their orders, have relatively smaller barriers, ranging from 1.22 to 0.76 eV. Overall, the [1,9] pathway has the lowest energies for all the transition barriers and intermediate states, while the [1,3] pathway has slightly lower transition barriers than the [1,2] pathway. Therefore, the [1,9] pathway is both thermodynamically and kinetically more favorable than the other two competing pathways.

## Discussion

On the basis of the experimental and theoretical results presented above, we finally remark on the new understandings of on-surface cyclodehydrogenation within the framework of pericyclic reactions. As shown above for the most favorable [1,9] pathway, we rationalized the first step of ring closure converting i to Int1 by a conrotatory electrocyclization and the second step of hydrogen migration across the fjord converting Int1 to Int2 by a [1,9]-sigmatropic hydrogen shift. The two pericyclic processes are both heat-induced and take place in-tandem. Another such example of pericyclic reactions is the formation of vitamin D_3_ in human skin, first by a photochemical electrocyclic ring opening and then by a thermal antarafacial [1,7]-sigmatropic hydrogen shift. Note that in the on-surface reactions studied in this work, the C_aryl_–C_aryl_ bond formation and the subsequent hydrogen shift do not just take place between two independent aryl rings, but instead between two aryl fragments linked by a C–C bridge located along the polymeric central axis. The dihedral angle between the neighboring anthrylene units is less than 90° even for the initial polymer state.^33^ Consequently, the two local atomic orbitals at the two bridge-head C atoms are not completely orthogonal to each other.^49^ As the neighboring anthrylene units rotate about the polymer axis to approach each other, the dihedral angle between them is reduced from that in the initial state i. After the new C_aryl_–C_aryl_ bond is formed at the fjord to connect the neighboring anthrylene units, the dihedral angle remains to be small in states Int1–Int3 and is further reduced in 1 as the aromaticity is restored on the molecular scaffold. Therefore, the π–π overlap between the bridge-head C atoms is enhanced during the cyclodehydrogenation processes, which can contribute to the cyclic conjugations in the molecular scaffold needed for the pericyclic reactions. After cyclodehydrogenation on one side is finished to give a half-polymer and half-ribbon hybrid structure,^32^ the situation may become even more pronounced for the cyclodehydrogenation on the other side of the polymer axis, which should be responsible for the previously reported lower energy cost for the second cyclodehydrogenation process than the first similar process on the two sides of the polymer axis, respectively.^36^

Despite the existence of cyclic conjugations during the C_aryl_–C_aryl_ bond formation and the subsequent hydrogen shift, there are still some differences between the cyclodehydrogenation reactions in the on-surface GNR synthesis and the classical pericyclic reactions. First and foremost, all studied on-surface cyclodehydrogenation reactions including the one in the present work take place in quasi-2D systems on coinage metal surfaces at high temperatures (see *T*_2_ = 670 K in Fig. 1). In comparison, the classical pericyclic reactions usually take place in solution phase at low-to-intermediate temperatures, all by going through the well-understood cyclic transition states, which can be considered as circular 1D conjugated structures. The rigidity of quasi-2D systems in contrast to the flexibility of 1D systems is noteworthy. The harsh condition as indicated by the high temperature needed for the on-surface reactions is related to the high theoretical energy cost (Fig. 4). Second, radical species can be formed in the on-surface cyclodehydrogenation processes. For instance, in the first step of electrocyclization, a biradicaloid intermediate Int1 is obtained with the C_aryl_–C_aryl_ bond formation, as indicated by the two dashed semicircles shown in Fig. 4a. In the second step of sigmatropic hydrogen shift, the biradicaloid Int1 is converted to a stable Kekulé structure Int2 in the most favorable [1,9] pathway. In comparison, in the less likely [1,2] and [1,3] pathways, an elimination of bottom-side D atom takes place in the second step giving a monoradical intermediate Int2′ as indicated by the single dashed semicircle in Fig. S15†, which then undergo [1,2] and [1,3] hydrogen shifts to form two other radical intermediates. As a result, Int2′ has a much higher energy than the stable Kekulé structure Int2. In comparison, radical species are rarely encountered in classical pericyclic reactions.^50^ And third, the coinage metal surface has two unique effects, which are weaker or even absent in the solution phase. On one hand, the substrate surface can stabilize the radical species formed during the on-surface reaction process by covalent interactions between radicals and substrate in addition to polarization effect.^35^ Without the stabilization effect of Au(111) surface, the energy costs for the entire cyclodehydrogenation process will be generally higher.^51^ On the other hand, the substrate can render steric hindrance and confinement effects on the intermediates. As a result, the conrotatory electrocyclization will be hampered, and the sigmatropic hydrogen shift will only take place for the top-side hydrogen in a suprafacial instead of antarafacial way. In comparison, classical pericyclic reactions usually take place in solution phase, where molecules have sufficient flexibilities *via* rotation and bending needed for the electrocyclization and suprafacial hydrogen shift (even antarafacial hydrogen shift is possible as seen in the vitamin D_3_ case). Despite all of these differences, the on-surface electrocyclization and sigmatropic hydrogen shift reactions are still concerted instead of stepwise. These on-surface reactions can be still placed under the umbrella of pericyclic reactions, only with a caveat that whether the renowned Woodward–Hoffmann symmetry rules well established for the classical pericyclic reactions are still applicable to the on-surface cyclodehydrogenations is an open question, which awaits the answer from future studies using many other quasi-2D systems that involve radical intermediates.

## Conclusion

In summary, our approach has combined selective deuteration, Raman spectroscopy, *in situ* mass spectrometry characterizations, and DFT and CI-NEB calculations to identify the cyclodehydrogenation pathway. In addition to previously proposed cyclodehydrogenation pathways in the literature, we have considered two more possibilities within the framework of pericyclic reactions. Both the [1,3] and [1,9] pathways involve a conrotatory electrocyclization and a suprafacial [1,3]- or [1,9]-sigmatropic hydrogen shift across the fjord where a new C_aryl_–C_aryl_ bond is formed. Since the final 7-aGNRs and gas-phase by-products are the same for the [1,3] and [1,9] pathways, the differentiation between them is solely based on theoretical calculations in the present work. In future studies, other experiments such as temperature-programmed desorption could be adopted to achieve additional information regarding the kinetics of the gas-phase by-products. This information on the time perspective may be used to further differentiate all possible reaction pathways with full certainty. Nevertheless, among these four mechanisms, the [1,9] pathway appears the most plausible. Our findings, along with recent studies of PAHs and particularly 7-aGNRs,^36,52^ suggest that the cyclodehydrogenation reactions can be considered within a more general class of pericyclic reactions.^53^ In this regard, the understanding acquired from this prototypical system should be applicable to other pericyclic and dehydrogenative reactions, such as the oxidative aromatic coupling and Scholl reaction,^1,2,8^ and sheds light on the recently reported cyclodehydrofluorination for PAH and GNR synthesis.^54,55^ This study also opens an avenue to achieving designer deuteration patterns in atomically precise GNRs and other large graphitic scaffolds, offering an isotopic labeling strategy to examine the dynamics of neutron scattering and nuclear magnetic resonance scattering.^45,56,57^

## Author contributions

A.-P. L. conceived the project and designed the experiments. J. H. and J. B. designed the theory tasks. C. M. and A.-P. L. performed the on-surface synthesis and the STM and mass spectrometry characterizations; P. V. B. and K. H. conducted molecule synthesis; C. M. and A. A. P. performed the Raman measurements; Z. X., L. L., J. H., and W. L. performed the theoretical calculations. C. M., J. H., and A.-P. L. wrote the paper with contributions from all authors.

## Conflicts of interest

There are no conflicts to declare.

## Supplementary Material

SC-012-D1SC04908A-s001
