# Supplementary material for: On-surface cyclodehydrogenation reaction pathway determined by selective molecular deuterations
Source: Chem Sci. 2021 Nov 16;12(47):15637–44. doi: 10.1039/d1sc04908a (PMC8653995; doi:10.1039/d1sc04908a)
Supplement: SC-012-D1SC04908A-s001 [file SC-012-D1SC04908A-s001.pdf]

Electronic Supplementary Material (ESI) for Chemical Science.
This journal is © The Royal Society of Chemistry 2021

*Electronic supplementary information for*

# On-surface cyclodehydrogenation Reaction Pathway Determined by Selective Molecular Deuterations

Chuanxu Ma,[ab] Zhongcan Xiao,[c] Peter V. Bonnesen,[a] Liangbo Liang,[a] Alexander A. Puretzky,[a] Jingsong Huang,[a]* Marek Kolmer,[ad] Bobby G. Sumpter,[a] Wenchang Lu,[ce] Kunlun Hong,[a] Jerzy Bernholc[ce] and An-Ping Li[a]*

[a]Center for Nanophase Materials Sciences, Oak Ridge National Laboratory, Oak Ridge, TN 37831, USA

[b]Hefei National Laboratory Center for Physical Sciences at the Microscale and Synergetic Innovation Center of Quantum Information & Quantum Physics, University of Science and Technology of China, Hefei, Anhui 230026, China

[c]Department of Physics, North Carolina State University, Raleigh, NC 27695, USA

[d]Ames Laboratory, U.S. Department of Energy, Ames, IA 50011, USA

[e]Computational Sciences and Engineering Division, Oak Ridge National Laboratory, Oak Ridge, TN 37831, USA

*Correspondence to: huangj3@ornl.gov; apli@ornl.gov

***Table of Contents:***

***Materials and Methods*................................................................S2-S6**

***Supplementary Text*.....................................................................S7-S9**

***Supplementary Figures and Table*.............................................S10-S28**

***References*...............................................................................S29-S30**

## 1. Materials and Methods

### 1.1 Synthesis of the deuterated 10,10'-dibromo-9,9'-bianthracene-1,1',4,4',5,5',8,8'-$d_8$ ($d_8$-DBBA)

The procedure is based on the superacid-catalyzed H/D exchange reactions on polycyclic aromatics described by Liang et al.[1] All reagents were used as received from the suppliers without further purification unless otherwise noted. 10,10'-dibromo-9,9'-bianthracene (DBBA) (>98% purity) and nonafluoro-1-butanesulfonic acid (>98% purity) were obtained from TCI America. $C_6D_6$ (99.8 atom% D) was obtained from Millipore-Sigma. In an argon-filled glovebox, a 15-mL heavy glass wall cylindrical pressure vessel was charged with a Teflon stir bar and DBBA (104 mg, 0.20 mmol). Benzene-$d_6$ (6.0 mL, 68 mmol) was added and the suspension swirled for several minutes to mostly dissolve the bianthracene. Nonafluoro-1-butanesulfonic acid (4 microliters, 0.025 mmol) was then added via Eppendorf pipet (*caution:* Nonafluoro-1-butanesulfonic acid is a super acid and should be handled with extreme care.) The vessel was then closed with a (back sealing) solid Teflon bushing with Viton O-ring, screwed in hand tight. The vessel was immersed about half-way into an oil bath and heated at 70 ºC for 2 days. The yellow-olive colored solution was allowed to cool to room temperature, then opened, and the reaction quenched by the addition of $D_2O$ (50 microliters). The product was isolated by adding 6 ml of saturated $NaHCO_3$ to the reaction mixture, followed by ca. 22 mL dichloromethane. The combined organic phase was separated from the aqueous layer and dried through a column of granular anhydrous $Na_2SO_4$. The solvents were removed by rotary evaporation to afford 107 mg of a micro-crystalline yellow solid. Proton and carbon Nuclear Magnetic Resonance (NMR) analysis on a 19.6 mg sample in $CDCl_3$ revealed partial deuteration of the 1,8 (ca. 23 atom% D) and 4,5 (ca. 62 atom% D) positions had taken place, with only trace exchange occurring at the 2,7 and 3,6 positions. The product was analytically pure.

The NMR sample was recovered and combined with the bulk intermediate product to afford 106 mg of combined material that was subjected to a second deuteration reaction. The material was again dissolved in 6.0 mL benzene-$d_6$ as above, but the amount of nonafluoro-1-butanesulfonic acid added was increased to 16.24 microliters (0.10 mmol). The vessel was sealed as described above and immersed about half-way into an oil bath and heated at 75 ºC for 3 days. Quenching and product isolation was performed as described above. After solvent removal, 107 mg of a yellow-green solid was obtained. Proton (60 second recycle delay) and carbon NMR analysis on a 10 mg sample in

$CDCl_3$ revealed nearly complete deuteration of the 1,8 (ca. 91 atom% D) and 4,5 (ca. 97 atom% D) positions had taken place, with only small amounts of exchange occurring at the 2,7 (<1 atom% D) and 3,6 (ca. 10 atom% D) positions (see Figs. S2–S5; percentages are relative to the essentially non-deuterated 2,7 position). $^1H$ NMR ($CDCl_3$): δ 8.72 (dd, $J_1$ = 9.1 Hz, $J_2$ = 1.1 Hz, 0.11 intensity/3 atom% H/97 atom% D, 4,4',5,5'); 7.79 (d, $J$ = 6.6 Hz, 3.60 intensity/90 atom% H/10 atom% D, 3,3',6,6'); 7.19 (d + s, $J$ = 6.6 Hz, 4.00 intensity/set to nominally 100 atom% H, 2,2',7,7'; singlet at 7.19 ppm at about 10% of total integral is from portion of 2,2',7,7' proton signal not coupled to the ca. 10 atom% D at 3,3',6,6' position); 7.09 (dd, $J_1$ = 8.8 Hz, $J_2$ = 1.3 Hz, 0.36 intensity/9 atom% H/91 atom% D, 1,1',8,8'). $^{13}C\{^1H\}$ NMR ($CDCl_3$): δ 133.3 (C9,9'); 132.3 (C"b"); 130.6 (C"a"); 128.0 (t, $J_{CD}$ = 24.8 Hz, C4,4',5,5'); 127.3 (C3,3',6,6'); 127.0 (t, $J_{CD}$ = 25.3 Hz, C1,1',8,8'); 126.4 (C2,2',7,7'); 124.0 (C10,10').

Proton and carbon NMR spectra were obtained on a Varian VNMRS 500 NMR spectrometer recorded at room temperature in $CDCl_3$ (7.27 ppm $^1H$ reference and 77.23 ppm $^{13}C$ reference). Carbon NMR spectra were obtained using inverse-gated decoupling with pw = 45 and a recycle delay of 20 seconds, and proton spectra were obtained using a 60 second recycle delay.

### 1.2 On-surface synthesis of the pristine and deuterated 7-aGNRs

The Au(111) single crystal was cleaned by repeated cycles of $Ar^+$ sputtering and annealing to 740 K. Pristine and deuterated DBBA molecules were well degassed and then evaporated from different cells onto the Au substrate, which was held at a temperature of 470 K. The coverage can be controlled by different deposition time with same deposition pressure and sample position. The samples were subsequently annealed at 470 K for 10 min and 670 K for 20 min, respectively, to induce polymerization and cyclodehydrogenation for the formation of pristine and deuterated 7-aGNRs.

### 1.3 STM/STS characterizations

STM characterizations were performed with a home-made variable temperature system at around 110 K under ultrahigh vacuum (UHV) conditions (better than $1\times10^{-10}$ torr) with a clean commercial PtIr tip. All STM images were acquired in a constant-current mode. The d$I$/d$V$ spectra were recorded using a lock-in amplifier with a sinusoidal modulation ($f$ = 1000 Hz, $V_{mod}$ = 20 mV) by turning off the feedback loop-gain. The polarity of the applied voltage refers to the sample bias

with respect to the tip.

### 1.4 Raman spectroscopy

Raman measurements, following our previous reports,[2, 3] were performed using a triple spectrometer (Jobin-Yvon T64000) consisting of a double monochromator coupled to a third monochromator stage with three 1800 grooves/mm gratings equipped with a liquid nitrogen-cooled CCD detector. All measurements were performed under a microscope in a backscattering configuration. A 532 nm laser (Excelsior, Spectra-Physics) light was focused onto the sample with a 100× objective (numeric aperture N/A = 0.9) to a spot size of about 1 μm. The excitation laser power on the sample was ~0.5 mW. The spectral resolution of the Raman spectrometer was ~0.7 $cm^{-1}$.

Some Raman spectra were acquired using a custom-built, high optical throughput setup using the same excitation laser and a microscope objective. In this case, the scattered Raman light was analyzed by a spectrometer (Spectra Pro 2300i, Acton, f = 0.3 m) that was coupled to a microscope and equipped with an 1800 groves/mm grating and a CCD camera (Pixis 256BR, Princeton Instruments).

### 1.5 Simulations of Raman spectra

First-principles DFT calculations were carried out using the VASP package,[4] where projector augmented-wave (PAW) pseudopotentials were used for electron-ion interactions and local density approximation (LDA) was adopted for exchange-correlation interactions. The kinetic energy cutoff for the plane wave was set at 500 eV. For the primitive unit cell of the 7-aGNRs, the lattice constant in the periodic direction ($x$ direction) is a = 4.256 Å. In the other two non-periodic directions, a vacuum region of about 18 Å was used to avoid spurious interactions with periodic images.[2] To simulate the isotope effect, a 2×1×1 supercell of 7-aGNRs was built to allow different hydrogen and deuterium patterns on the edges to be studied. Gamma-centered 24×1×1 and 12×1×1 k-point samplings were used for the primitive cell and supercell, respectively. All atoms were relaxed until the residual forces were below 0.001 eV/Å.

Based on the fully optimized structures, phonon calculations were performed using the finite difference scheme implemented in the Phonopy software.[5] Hellmann-Feynman forces were computed by VASP for both positive and negative atomic displacements ($\delta$ = 0.03 Å), and then used in Phonopy to construct the dynamic matrix. The diagonalization of the dynamic matrix gives

phonon frequencies and phonon eigenvectors (i.e., atomic vibrations). Raman intensities of phonon modes were then calculated using the in-house developed Raman modeling package.[6, 7] The computation of Raman intensity essentially requires the derivatives of the dielectric tensor $\varepsilon_{\alpha\beta}(E_{\rm L})$ with respect to atomic displacements, which can be achieved by the finite difference method as well. For both positive and negative atomic displacements ($\delta$ = 0.03 Å), the frequency-dependent dielectric tensors $\varepsilon_{\alpha\beta}(E_{\rm L})$ were computed by VASP and then their derivatives can be obtained. Note that the dielectric tensor $\varepsilon_{\alpha\beta}(E_{\rm L})$ should be calculated at the incident laser photon energy $E_{\rm L}$. Since DFT often underestimates the optical gaps (for 7-aGNRs, the experimental optical gap is about 2.1 eV (ref. [8]), while the DFT bandgap is 1.62 eV), a scaling factor was needed. The experimental laser photon energy is 2.33 eV in this work, so the value of the incident laser photon energy used in our Raman calculations should be around 2.33×(1.62÷2.1)=1.8 eV. Finally, based on the phonon frequencies, phonon eigenvectors, and the derivatives of dielectric tensors, Raman tensor $\tilde{R}$ of any phonon mode can be obtained. In the experimental back-scattering configuration, the polarization vectors of the incoming and scattered light are in the *x*-*y* plane. Averaging over all possible in-plane polarizations, the Raman intensity of any phonon mode is given by $I \propto \frac{1}{4}\frac{n_j+1}{\omega_j}(|\tilde{R}_{11}|^2+|\tilde{R}_{12}|^2+|\tilde{R}_{21}|^2+|\tilde{R}_{22}|^2)$ (ref. [7]). With the calculated Raman intensities *I*(*j*) and phonon frequencies $\omega_j$, the Raman spectrum can be obtained after Lorentzian broadening. For the spectra in Fig. 2b, a Lorentzian broadening of 16 $cm^{-1}$ was used, which shows a clear splitting of the BLM3.

From the simulated Raman spectrum with a smaller broadening (2 $cm^{-1}$), one can find that the BLM is also split, with a smaller splitting of about 7.7 $cm^{-1}$, compared to the larger splitting of about 19.7 $cm^{-1}$ of the BLM3, but the SLM does not split (Fig. S8). While there is no apparent splitting for the BLM in experiment, a slight broadening is indeed observed with high-resolution Raman spectroscopy when comparing the pristine and deuterated GNRs (see Fig. S9). This could be assigned to two reasons. First, the splitting is overestimated in the simulation. For example, the experimentally observed splitting of BLM3 is about 13.0 $cm^{-1}$, which is smaller than the splitting of 19.7 $cm^{-1}$ from the simulation. Therefore, the experimental splitting of the BLM should be smaller than the calculated value of 7.7 $cm^{-1}$. Second, the low percentage of isotopic impurity may slightly

modify the H/D patterns, which would further suppress the BLM splitting.

### 1.6 Mass spectrometry

A commercial RGA100 residual gas analyzer (Stanford Research Systems) was used. By using the O100MAX Maximum Insertion nipple, a short sample-ionizer length of about 2 cm was achieved and used for all mass spectrometry measurements in experiment. Each time the RGA100 is fully degassed before recording the signal. The polymers were further annealed at 520 K for 1 h to fully remove the residual bromine (Br) atoms generated during polymerization[9] (Fig. S10). Coverage was controlled to be lower than one monolayer (around 0.5~0.9 monolayer) to avoid influences from the multiple layer molecular precursors[10] (Fig. S12). Then the mass spectra of HD (3 amu), $D_2$ (4 amu), $H_2$ (2 amu), and HBr (81 amu) were recorded at annealing of 670 K for at least 25 min. As reference, only $H_2$ are detected without HD and $D_2$ for pristine precursors (Fig. S10). $H_2$, HD and $D_2$ signals were also measured for the clean Au substrate as background (Fig. S11).

### 1.7 CI-NEB simulations

The first-principles density functional theory (DFT) calculations for energy profiles were performed with the Quantum Espresso code,[11] using ultrasoft pseudopotentials[12] and Perdew-Burke-Ernzerhof (PBE) exchange-correlation functional.[13] The kinetic energy cutoff for the plane wave basis of Kohn-Sham wavefunctions was set at 24 Ry, and that for the charge density at 200 Ry. The supercells consist of four anthrylene units with periodic boundary condition, which were relaxed until forces on atoms reached a threshold of 0.026 eV/Å. The periodic direction of the polymer is aligned along the [110] direction of Au to allow for minimum lattice mismatch and strain. A non-local van der Waals (vdW) correction,[14] i.e., the self-consistent vdW-DF method, as used in our previous report,[15] was chosen to calculate energies of the polymer and the GNR on the metal substrate. The energy barriers of the reaction were calculated using the climbing image nudged elastic band (CI-NEB) method.[16] The forces on images were relaxed until they reached a threshold of 0.1 eV/Å. Different reaction pathways involving [1,2], [1,3]-sigmatropic, and [1,9]-sigmatropic hydrogen shift were examined (Fig. S15).

## 2. *Supplementary Text*

## 2.1 The Four different cyclodehydrogenation pathways discussed in this work

The direct hydrogen elimination pathway proposed by Björk et al.[17] is shown in Fig. S1a. From the initial state **i**, the neighboring anthrylene units first rotate about the polymer axis to approach each other, allowing two benzyne groups ($C_6H_2D_2$) on the same side of the polymer to form a single $C_{sp^3}$–$C_{sp^3}$ bond, giving **Int1**, with the two D atoms both facing down to the Au surface. Then, the two D atoms simultaneously eliminate to the Au surface, giving state **1**. States **2**, **3**, and **4** are obtained after the formation of subsequent three C–C bonds in a domino-like fashion. Final state **f** obtained following this pathway is pristine 7-aGNRs without deuteration.

The [1,2] pathway proposed by Blankenburg et al.[18] is shown in Fig. S1b. The neighboring anthrylene units in the initial state **i** first rotate about the polymer axis to approach each other, allowing two benzyne groups ($C_6H_2D_2$) on the same side of the polymer to form a single $C_{sp^3}$–$C_{sp^3}$ bond, giving **Int1**. However, unlike the Björk pathway (Fig. S1a), the two D atoms respectively face down to the Au surface (for the one on the tilting-down anthrylene unit) and up to the vacuum side (for the one on the tilting-up anthrylene unit). Then the D atom on the bottom side of the newly formed bond eliminates to the Au surface, giving **Int2**. Following that, the D on the top side migrates by a [1,2] hydrogen shift to the neighboring edge C atom of the same anthrylene unit, as highlighted by the red arrow and line segment, giving **Int3**. Another C–C bond forms with the neighboring anthrylene unit on the right with similar processes in a one-side domino-like fashion, respectively giving **Int4**, **Int5**, and **Int6**. After the desorption of two neighboring H atoms to the Au surface, state **1** is formed. With these repeating steps, deuterated 7-aGNRs (state **f**) forms with an alternating H/D pattern on the edges.

The [1,9] pathway proposed in this work based on the [1,9]-sigmatropic hydrogen shift is shown in Fig. S1c. First of all, **Int1** is formed in the same way as in the [1,2] pathway. Following that, the D atom on the top side of the newly formed bond migrates by a [1,9]-sigmatropic hydrogen shift across the fjord to an edge C atom of a neighboring anthrylene unit, as highlighted by the red arrow and line segments, giving **Int2**. Next, the D atom at the other bonding C and the H atom at the edge C, both facing down to the Au substrate, are sequentially eliminated as ad-atoms on the Au surface, respectively giving **Int3** and state **1**, thereby restoring the aromaticity of the scaffold. Subsequently, a neighboring $C_{aryl}$–$C_{aryl}$ bond forms in the same way as in a one-side domino-like mechanism,[18-20]

giving state **2** with two D atoms located at the previously down-tilted anthrylene edge. Finally, the other side of the polymer goes through the same reaction path, which leads to state **f** with the H/D pattern as identified by Raman spectroscopy and DFT calculations.

Two different [1,3] pathways, based on the suprafacial and antarafacial [1,3]-sigmatropic H shift, are shown as Figs. S1$d_1$ and S1$d_2$, respectively. First of all, **Int1** and **Int2** are formed in the same way as in the [1,2] pathway. However, in the following step the D atom on the top side of the newly formed bond migrates by a suprafacial or antarafacial [1,3]-sigmatropic hydrogen shift across the fjord to the front or rear side of an edge C atom of a neighboring anthrylene unit, giving **Int3** of different structures, with the previous H atom on the edge being pushed down or up, respectively. Then the H or D atoms on the edge facing down to the Au substrate are eliminated, giving **1** with different isotopes on the edge, thereby restoring the aromaticity of the scaffold. Subsequently, a neighboring $C_{aryl}$–$C_{aryl}$ bond forms in the same way as in a one-side domino-like mechanism,[18-20] giving state **2** with two D or two H atoms located at the previously down-tilted anthrylene edge. Finally, the other side of the polymer goes through the same reaction path, which leads to state **f**. The suprafacial [1,3] pathway produces the same H/D pattern as the [1,9] pathway, whereas the antarafacial [1,3] pathway gives a pristine 7-aGNR.

The same H/D pattern in the final deuterated 7-aGNR can be achieved in the [1,2], [1,9] and suprafacial [1,3] pathways, while the pristine 7-aGNR can be achieved in the direct hydrogen elimination and the antarafacial [1,3] pathways.

## 2.2 D elimination and migration order in different pathways

In the direct elimination pathway (Fig. S1a), D atoms will be directly eliminated after the $C_{sp^3}$–$C_{sp^3}$ bond formation without undergoing migration. In both the [1,2] pathway (Fig. S1b) and [1,3] pathway (Fig. S1d), the D facing down to the Au surface will first eliminate after the $C_{sp^3}$–$C_{sp^3}$ bond formation, and then the other D facing up to the vacuum side will migrate between two immediate neighboring C atoms (thus the term of [1,2] shift) or across the fjord to the edge of a neighboring anthrylene unit, respectively. Differently, in the [1,9] pathway, the D facing up to the vacuum will migrate first also across the fjord to the edge of a neighboring anthrylene unit, while the D facing

down to the Au surface remains attached on the intermediate structure. The terms of [1,3] and [1,9] pathways are named according to the order terms of respective [1,3]- and [1,9]-sigmatropic hydrogen shifts.

### 2.3 Estimated gas-phase products in different pathways

In the direct elimination pathway,[17] the two D atoms at the two bonding C atoms are eliminated to form $D_2$ only (see Fig. S1a). In the [1,2] pathway,[18] two immediately neighboring H atoms on the ribbon edge are eliminated to give prevailing $H_2$, leaving the well-separated D atoms to predominantly form $D_2$ (see Fig. S1b). In the [1,3] pathway, HD will be predominantly formed with a suprafacial [1,3]-sigmatropic hydrogen shift, while only $D_2$ will be generated with an antarafacial [1,3]-sigmatropic hydrogen shift (see Fig. S1d). Differently, as shown in Fig. S1c and Fig. 4a in the main text, in the [1,9] pathway, the elimination of D and H atoms happens sequentially and has a lateral separation of only one C–C bond. While full scrambling of the eliminated H and D atoms will give a $H_2$/HD/$D_2$ ratio of exact 1:2:1 (ref. [21]), the proximity between the eliminated H and D atoms suggests the more preferable formation of HD, thereby giving an expected HD ratio larger than 2.

The exact ratio between $H_2$, HD and $D_2$ cannot be predicted due to two origins. The main reason is that the molecular precursors are not 100% isotopically pure. The other reason is that the kinetic isotope effect could slightly affect the ratio. However, one can still expect the dominant gas-phase products in different pathways, such as, dominant $D_2$ from direct elimination pathway,[17] dominant $H_2$ and $D_2$ from the [1,2] pathway,[18] dominant HD from the suprafacial [1,3] pathway, dominant $D_2$ from the antarafacial [1,3] pathway, and dominant HD from the [1,9] pathway, as illustrated and summarized in Fig. S13.

## 3. Supplementary Figures and Table

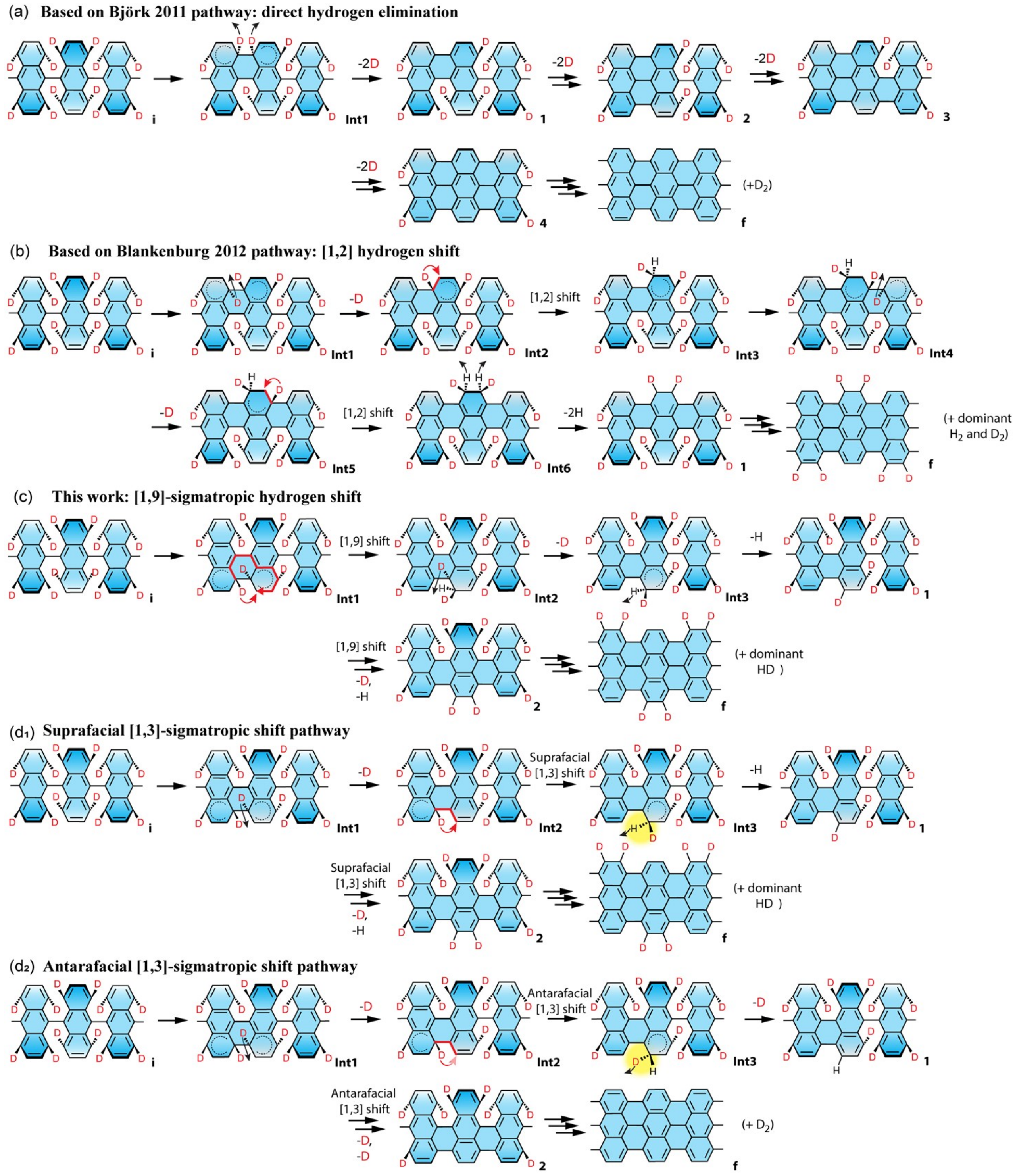


**Fig. S1** Four different cyclodehydrogenation pathways discussed in this work. (a) Cyclodehydrogenation based on the pathway proposed by Björk et al.[17] In this pathway, the gas product will be only $D_2$. (b) Cyclodehydrogenation based on the pathway proposed by Blankenburg

et al.,[18] namely the [1,2] pathway. According to the elimination sequence for the D and H atoms, one can expect that the dominant gas-phase products will be $H_2$ and $D_2$. (c) Cyclodehydrogenation proposed in this work based on the [1,9]-sigmatropic hydrogen shift, namely the [1,9] pathway. According to the elimination sequence for the D and H atoms, one can expect that the dominant gas-phase products will be HD. ($d_1$) Cyclodehydrogenation pathway based on the suprafacial and ($d_2$) antarafacial [1,3]-sigmatropic H shift, namely [1,3] pathways, with dominant gas-phase products of HD and $D_2$, respectively. Our CI-NEB simulation indicates that the antarafacial [1,3]-sigmatropic pathway has an extremely high energy barrier of about 3 eV with respect to **Int2**. Raman spectroscopy and in-situ mass spectrometry results can exclude the Björk pathway in (a) and the antarafacial [1,3] pathway in ($d_2$). Therefore, in the text we mainly focus on the [1,2], [1,3] and [1,9] pathways, where the latter two involving [1,3]- and [1,9]-sigmatropic hydrogen shifts are referred to as *suprafacial* pathways following the terminologies of pericyclic reactions.

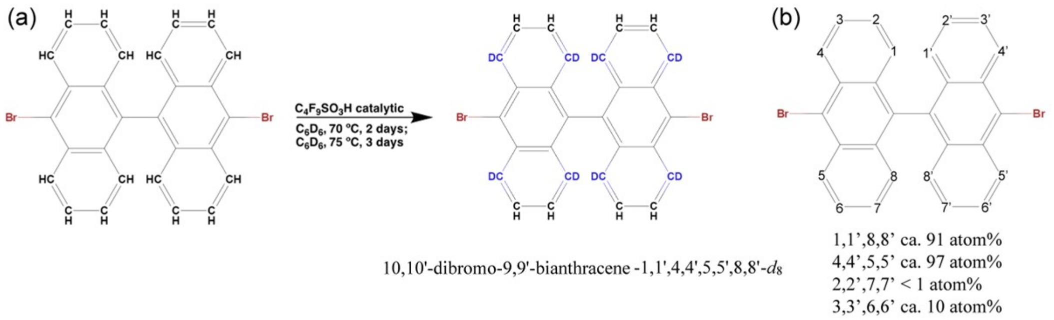


**Fig. S2** Scheme of synthesis of the site-specific deuterated DBBA precursors. (a) Scheme of the superacid-catalyzed partial H/D exchange of DBBA to form 10,10'-dibromo-9,9'-bianthracene-1,1',4,4',5,5',8,8'-$d_8$ ($d_8$-DBBA). (b) The atom% D in each site with an estimated uncertainty of $\leq \pm$ 1%.

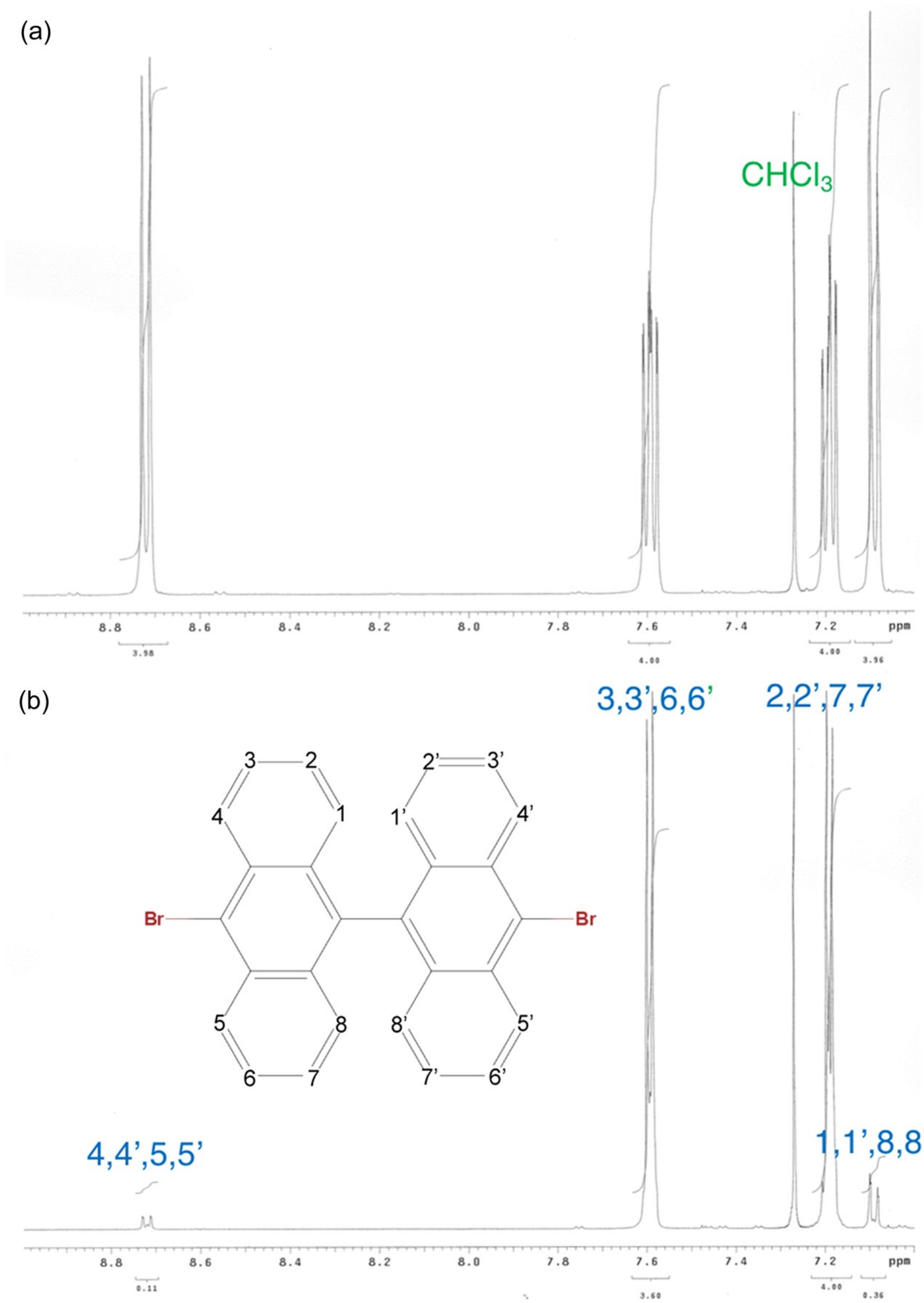


**Fig. S3** $^1$H NMR spectra. (a) DBBA (60 second recycle delay). (b) 10,10'-dibromo-9,9'-bianthracene-1,1',4,4',5,5',8,8'-$d_8$.

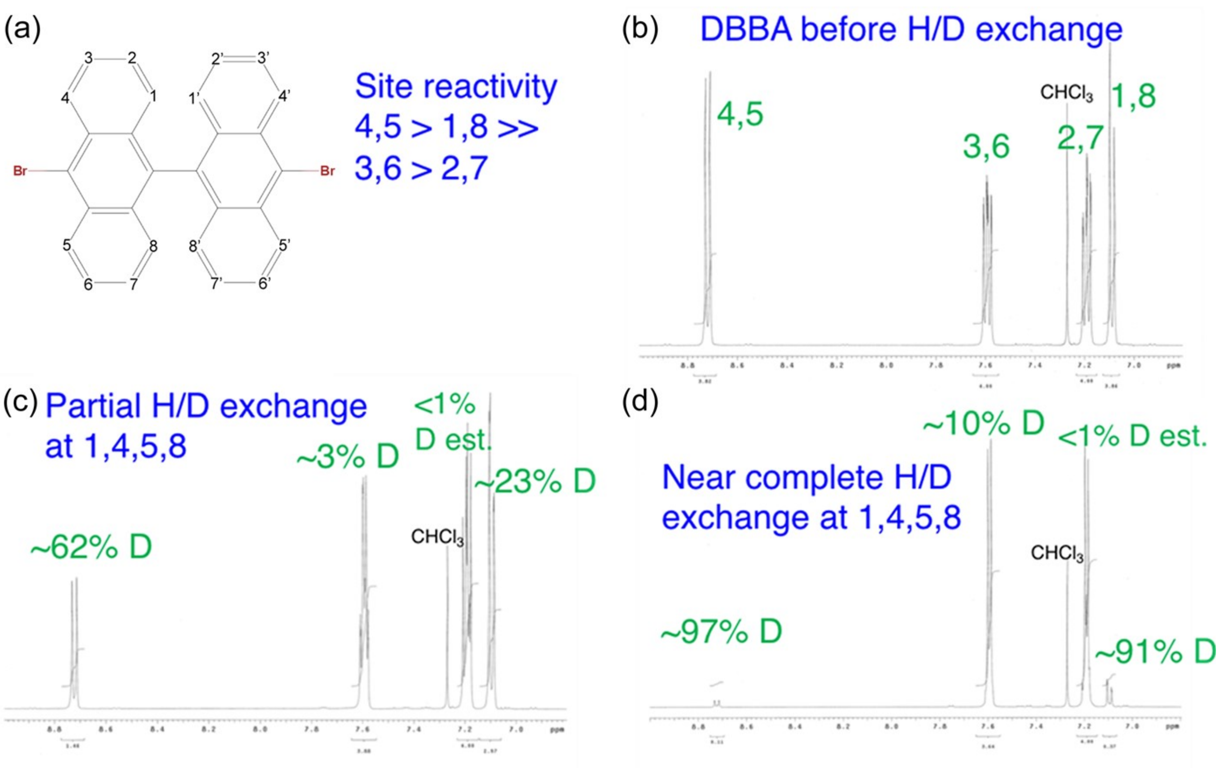


**Fig. S4** Conversion of DBBA to 10,10'-dibromo-9,9'-bianthracene-1,1',4,4',5,5',8,8'-$d_8$ as evaluated by $^1$H NMR in $CDCl_3$. (a) Determined site deuteration reactivity in DBBA. (b) $^1$H NMR of DBBA before exchange, (c) after partial exchange at 1,4,5,8 positions (70 ºC for 2 days), and (d) near full exchange at 1,4,5,8 positions (additional reaction at 75 ºC for 3 days).

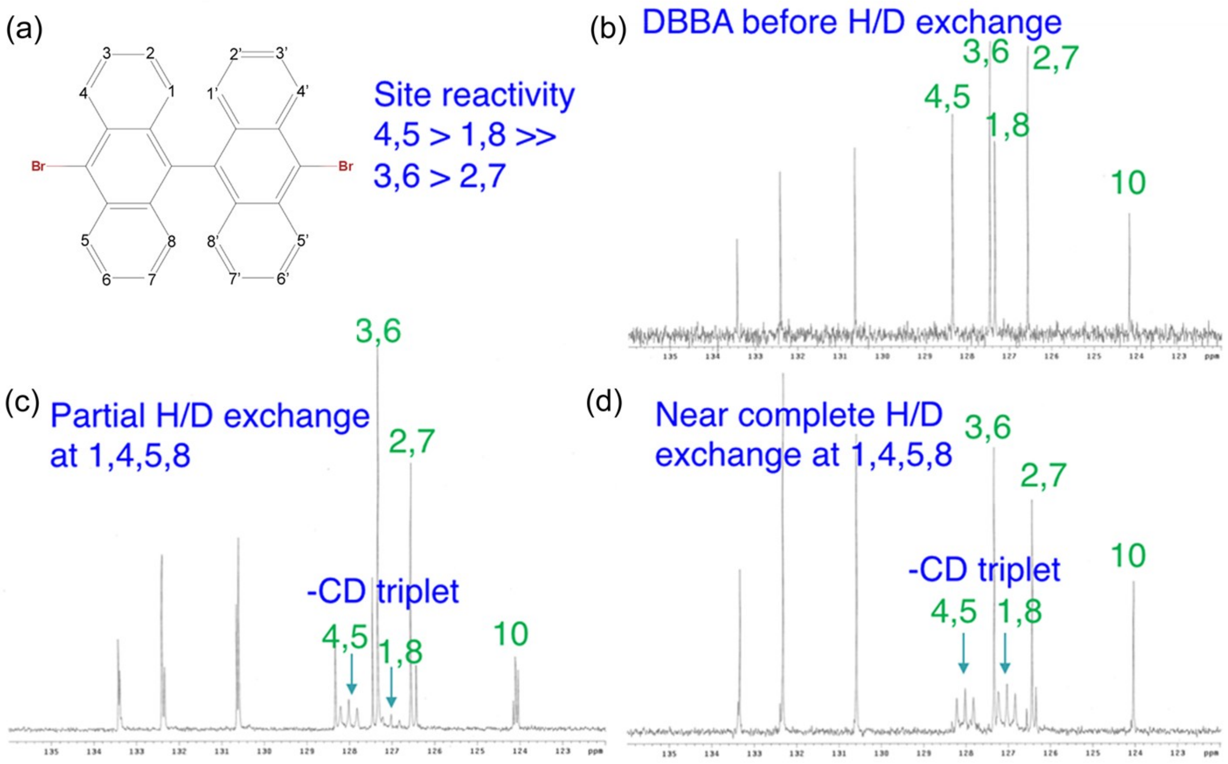


**Fig. S5** Conversion of DBBA to 10,10'-dibromo-9,9'-bianthracene-1,1',4,4',5,5',8,8'-$d_8$ as evaluated by $^{13}$C NMR in $CDCl_3$. (a) Determined site deuteration reactivity in DBBA. (b) $^{13}$C NMR of DBBA before exchange, (c) after partial exchange at 1,4,5,8 positions (70 ºC for 2 days), and (d) near full exchange at 1,4,5,8 positions (additional reaction at 75 ºC for 3 days).

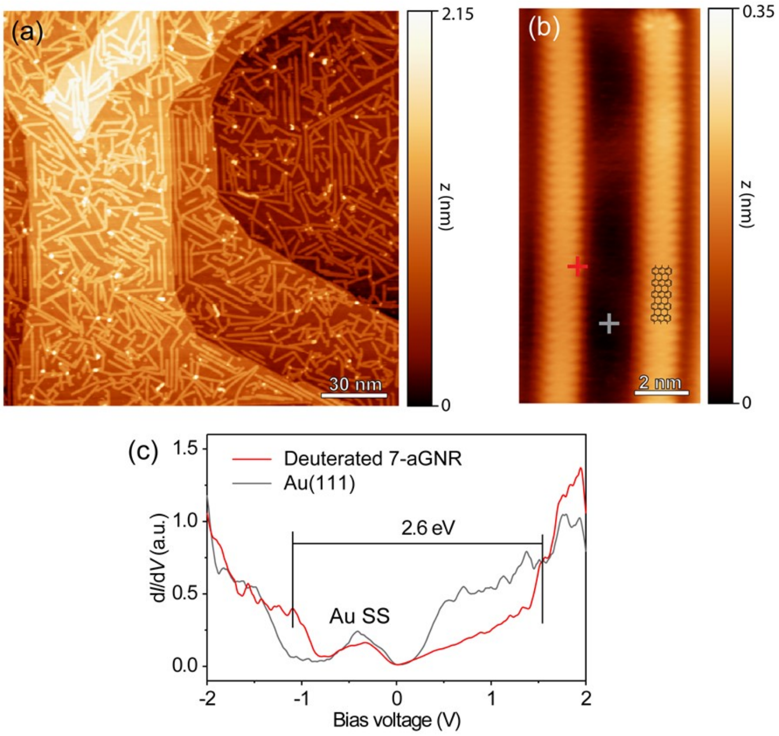


**Fig. S6** Structural and electronic properties of the deuterated 7-aGNRs. (a) Large-area STM topographic image of the deuterated 7-aGNRs (sample bias $V_s = -2$ V, tunneling current $I_t = 10$ pA). (b) High-resolution STM image of two deuterated 7-aGNRs, with the structural model superimposed ($V_s = -0.3$ V, $I_t = 70$ pA). (c) d$I$/d$V$ curves acquired at the red and grey crosses marked sites in (b), respectively, showing a bandgap of 2.6 eV with the valence and conductance band peaks at −1.1 and 1.5 V for the deuterated GNR ($V_s = -2$ V, $I_t = 100$ pA). The electronic states originated from the Au surface states are also marked. Both the atomic image and the bandgap are similar to those in pristine 7-aGNRs.[2, 19, 20, 22, 23]

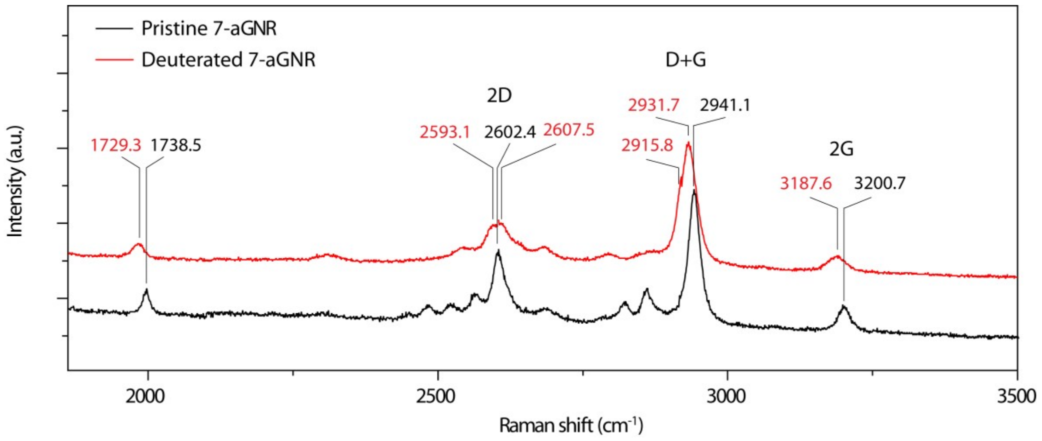


**Fig. S7** High frequency Raman spectra of the pristine and deuterated 7-aGNRs. Clear redshift, splitting, and broadening for the Raman modes can be seen in the spectrum acquired from the deuterated 7-aGNR with respect to its pristine counterpart. The corresponding peaks can be assigned to overtones of D and G bands and their combinations, i.e., 2D, D+G, and 2G, similarly to the previous report.[24]

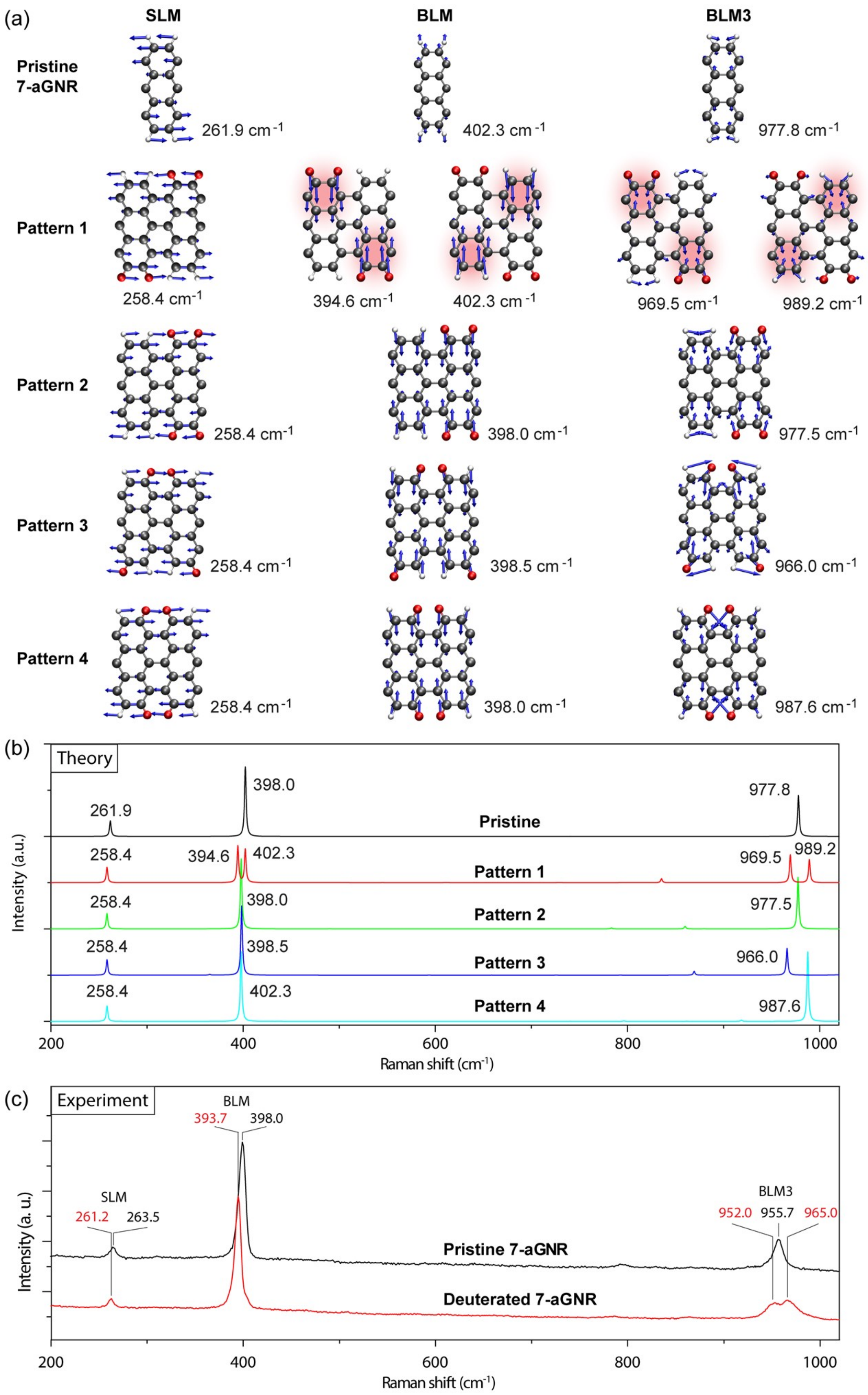


**Fig. S8** Simulated Raman vibrational modes for pristine and deuterated 7-aGNRs with different H/D

patterns. (a) Shear-like mode (SLM) and two breathing-like modes (BLM and BLM3) patterns of the pristine and deuterated 7-aGNRs with four different types of H/D patterns (H: white, D: red). The deuterated 7-aGNRs have supercells that double the unit cell of pristine 7-aGNR. Only pattern 1 shows the splitting of the BLM and BLM3. (b) Simulated Raman spectra for the pristine and deuterated GNRs with H/D patterns 1−4. The data were obtained with a smaller broadening (2 $cm^{-1}$), compared to those in Fig. 2b (16 $cm^{-1}$). Only pattern 1 shows splitting for BLM and BLM3, while SLM does not for all patterns. For pattern 1, the BLM's splitting is about 7.7 $cm^{-1}$, much smaller than that for BLM3 (19.7 $cm^{-1}$). In the experiment, we indeed observe a broadened BLM in deuterated GNRs compared to that in the pristine GNR (Fig. S9). (c) Experimental Raman spectra of the pristine and deuterated GNRs, same as those in Fig. 2a but with a narrow frequency range, for comparison with (b).

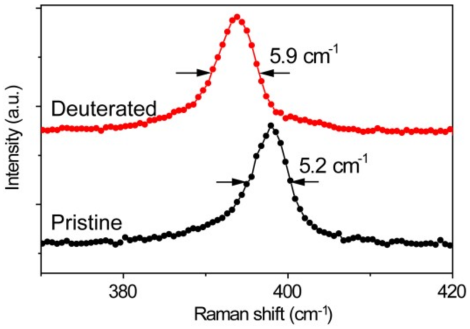


**Fig. S9** Slight broadening in high-resolution Raman spectra for the BLM in the deuterated GNRs compared to the pristine one. Two representative experimental Raman spectra showing the BLM in the pristine (black) and deuterated (red) GNRs. Compared with the BLM in the pristine 7-aGNR, the same mode in the deuterated ones shows a slight broadening of about 0.7 $cm^{-1}$, together with the expected redshift.

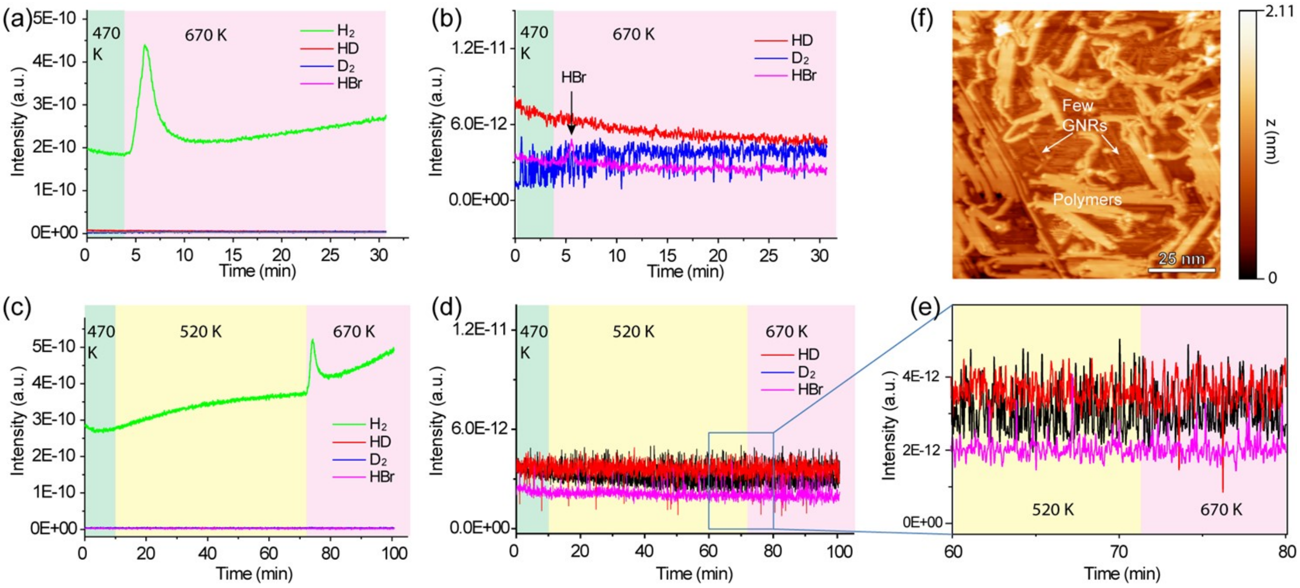


**Fig. S10** Mass spectrometry study of the pristine polymers during different annealing temperatures. (a) Mass spectra of $H_2$ (2 amu), HD (3 amu), $D_2$ (4 amu) and HBr (81 amu) during annealing of the polymer intermediate from pristine DBBA precursor molecules on Au(111) at 470 and 670 K, as marked with shadowing. The $H_2$ background of about 2~3E$^{-10}$ a.u. comes from the residual hydrogen in the chamber. The $H_2$ background keeps increasing during sample annealing due to the continuous degassing of the filament, sample holder, Au substrate, and surroundings (see Fig. S11). (b) The same mass spectra as (a) but at a zoomed-in scale. A small peak of HBr (marked with a black arrow) is observed. No signal is observed for HD and $D_2$. The area-based ratio between $H_2$ and HBr is estimated to be about 560:1. If all the Br atoms leave the Au surface as HBr, the ratio should be around 3:2. This result indicates that most Br atoms (>99%) do not desorb from the Au surface as HBr. Since $Br_2$ was not observed in previous experiments,[9, 10] we propose the desorption of Br in other compositions, such as AuBr, as suggested in previous reports of metal halides detected from various metal surfaces.[9, 25-27] (c)−(e) Mass spectra of $H_2$, HD, $D_2$ and HBr during annealing of the polymer intermediate from pristine DBBA molecules on Au(111) with an additional annealing step at 520 K for 1 h. The HBr is not observed anymore after increasing the temperature to 670 K. Note that with pristine DBBA precursors, no HD and $D_2$ signals are detected, but only $H_2$. (f) STM image of the polymers after annealed at 520 K for 1 h ($V_s = -2$ V, $I_t = 2$ pA). Only few polymers, marked with white arrows, are converted to GNRs.

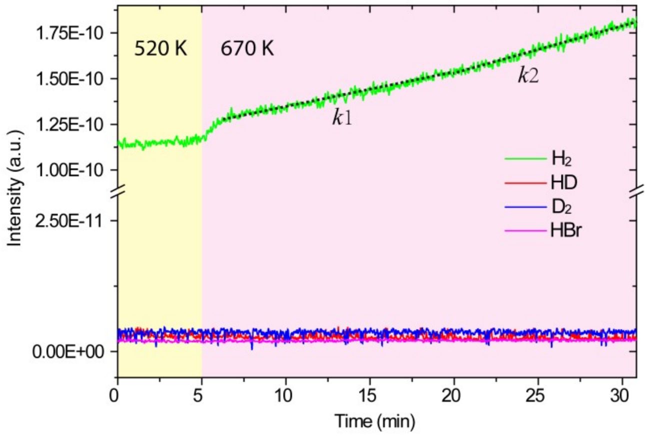


**Fig. S11** Mass spectra of $H_2$, HD, $D_2$ and HBr during annealing of the clean Au(111) substrate at 520 and 670 K. No signal increase of HD, $D_2$, and HBr is observed. For $H_2$, a small increase step (about $1.5E^{-11}$ a.u.) is observed right after annealing for 670 K. Then a continuous increase of $H_2$ occurs, with two almost linear parts of slightly different slopes before and after 20 min.

| (a) | 1 | 2 | 3 | 4 | 5 | 6 |
|---|---|---|---|---|---|---|
| Coverage (monolayer) | 0.9 | 0.86 | 0.86 | 0.78 | 0.78 | 0.55 |
| $HD:D_2$ | 2.67 | 2.69 | 2.77 | 2.70 | 2.70 | 2.68 |
| $H_2:D_2$ | 1.41 | 1.20 | 1.19 | 1.14 | 1.24 | 1.31 |
| D/H ratio | 85.1% | 92.6% | 92.1% | 94.4% | 90.7% | 88.3% |

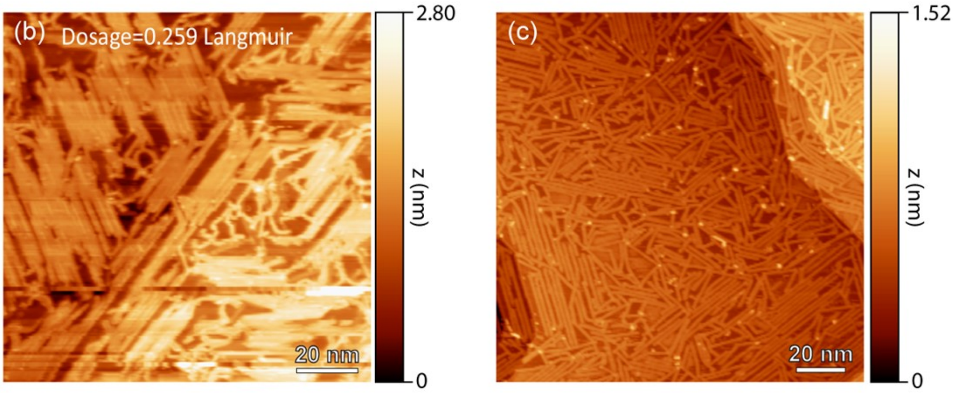


**Fig. S12** Measured $HD:D_2$ and $H_2:D_2$ ratios by mass spectrometry from samples with different coverage (<1 monolayer) of GNRs. (a) $HD:D_2$ and $H_2:D_2$ ratios, and obtained D/H ratios, for samples grown with different coverages. (b) STM image of the deuterated polymer, and (c) sequentially converted 7-aGNR sample with coverage of about 0.9 monolayer. Based on the results of the six measurements in (a), averaged $H_2:HD:D_2$ ratios can be obtained as 1.25:2.70:1, giving a D/H ratio of about 90.4%, which is slightly lower than the expected value (~98%) according to the isotopic purity. This could be attributed to the enhanced ratio of $H_2$, due to the kinetic isotopic effect during the formation of the gas-phase products on Au surface or the surrounding background (Fig. S11), which makes the estimation of the exact ratio of $H_2$ hard.

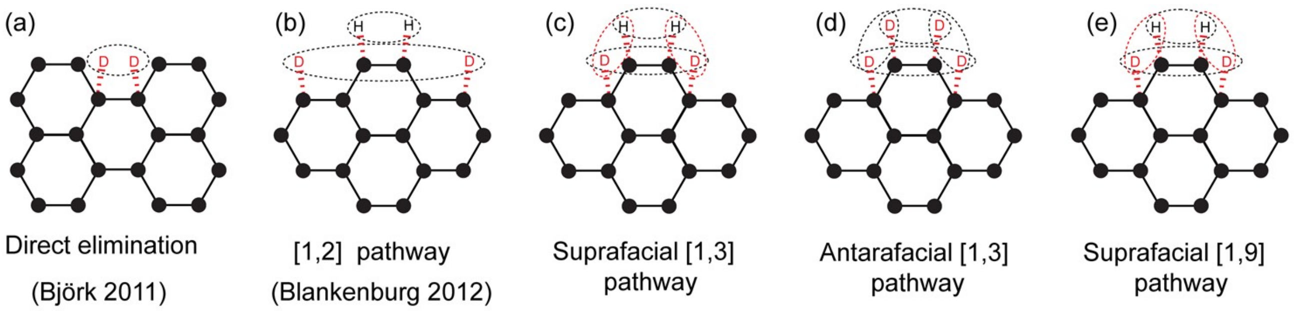


(f)

| Pathway | Direct elimination | [1,2] pathway | *Suprafacial* [1,3] pathway | *Antarafacial* [1,3] pathway | *Suprafacial* [1,9] pathway |
|---|---|---|---|---|---|
| Gas - phase products | Only $D_2$ | $H_2$ and $D_2$ | $H_2$ , HD and $D_2$ ; HD dominant | Only $D_2$ | $H_2$ , HD and $D_2$ ; HD dominant |

**Fig. S13** Gas-phase products expected from different reaction pathways with $d_8$-DBBA precursors. (a) Schematics of the eliminated D atoms' spatial locations with respect to the lattice of graphene (the black hexagons with black spots representing C atoms) in the direct elimination pathway (Fig. S1a), referring to the step from **Int1** to state **1**. (b) Schematics of the eliminated D and H atoms' spatial locations with respect to the lattice of graphene (the dashed hexagons) in the [1,2] pathway (Fig. S1b), referring to the steps from **Int1** to **Int2**, **Int4** to **Int5**, and **Int6** to state **1**. (c) Schematics of the eliminated D and H atoms' spatial locations with respect to the lattice of graphene (the dashed hexagons) in the suprafacial [1,3] pathway (Fig. S1$d_1$), referring to the steps from **Int1** to **Int2**, **Int3** to state **1**, and state **1** to state **2**. (d) Schematics of the eliminated D and H atoms' spatial locations with respect to the lattice of graphene (the dashed hexagons) in the antarafacial [1,3] pathway (Fig. S1$d_2$), referring to the steps from **Int1** to **Int2**, **Int3** to state **1**, and state **1** to state **2**. (e) Schematics of the eliminated D and H atoms' spatial locations with respect to the lattice of graphene (the dashed hexagons) in the suprafacial [1,9] pathway (Fig. S1c and Fig. 4a), referring to the steps from **Int2** to **Int3**, **Int3** to state **1**, and state **1** to state **2**. The possible recombination of the eliminated H/D is labeled, with black ovals for $H_2$ and $D_2$, and red ovals for HD. Note that different graphene lattice fragments are used due to the distinct sites of the eliminated hydrogen (H/D) in different pathways. (f) Expected gas-phase products from different pathways. Here the elimination sequence is also considered.

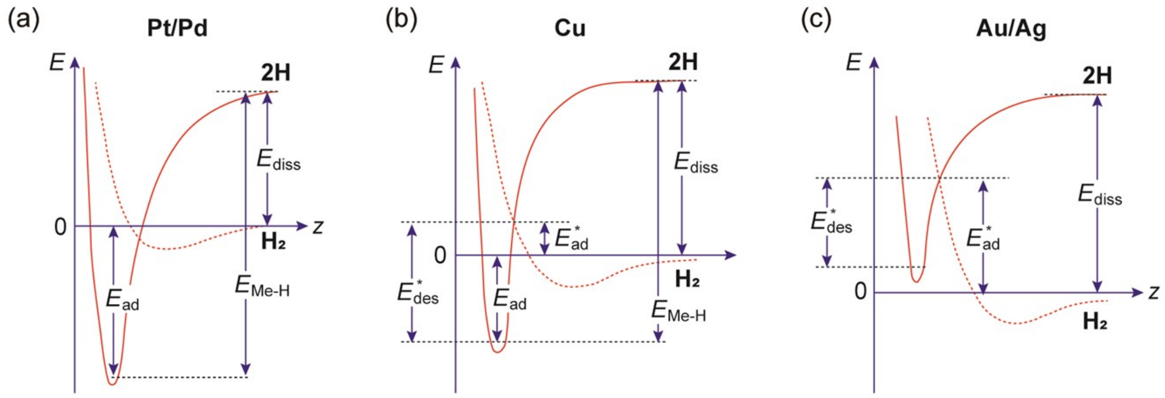


**Fig. S14** Schematic drawings of one-dimensional Lennard-Jones potential energy diagrams for molecular physisorption as indicated by dashed curves (Me+$H_2$) and dissociative chemisorption as indicated by solid curves (Me+2H) of hydrogen on different metal (Me) surfaces.[28] (a) Spontaneous dissociation on Pt and Pd, (b) activated dissociation on Cu with chemisorption being more favorable than physisorption, and (c) activated dissociation on Au and Ag with physisorption being more favorable than chemisorption, where $E_{ad}^*$ in (b,c) is the barrier height for dissociation with respect to free $H_2$ molecule, $E_{des}^*$ in (b,c) is the barrier height for combinative desorption from chemisorbed H atoms to $H_2$ molecule, $E_{ad}$ in (a,b) is the chemisorption energy of H atoms, $E_{diss}$ in (a-c) is the dissociation energy of free $H_2$ molecule to free H atoms, and $E_{Me\text{-}H}$ in (a,b) is the energy of the Me-H bond. Note that the depth of the chemisorption potential is above $E = 0$ for the cases of Au and Ag in (c), which makes the combinative desorption of two H atoms as $H_2$ an exothermic and preferable process. Therefore, during the cyclodehydrogenation process, the eliminated H/D atoms prefer a combinative desorption from Au surface, especially when the $H_2$ molecules are constantly pumped away by the ion pump that maintains the chamber under a UHV condition (better than $1\times10^{-10}$ torr). This is in contrast to the scenario as revealed in ref. [29], where $H_2/D_2$ molecules of various partial pressures ($10^{-5} \sim 10^{-1}$ torr) are present, and therefore the hydrogen molecules can undergo an endothermic chemisorption at elevated temperatures, following the Le Chatelier's principle.

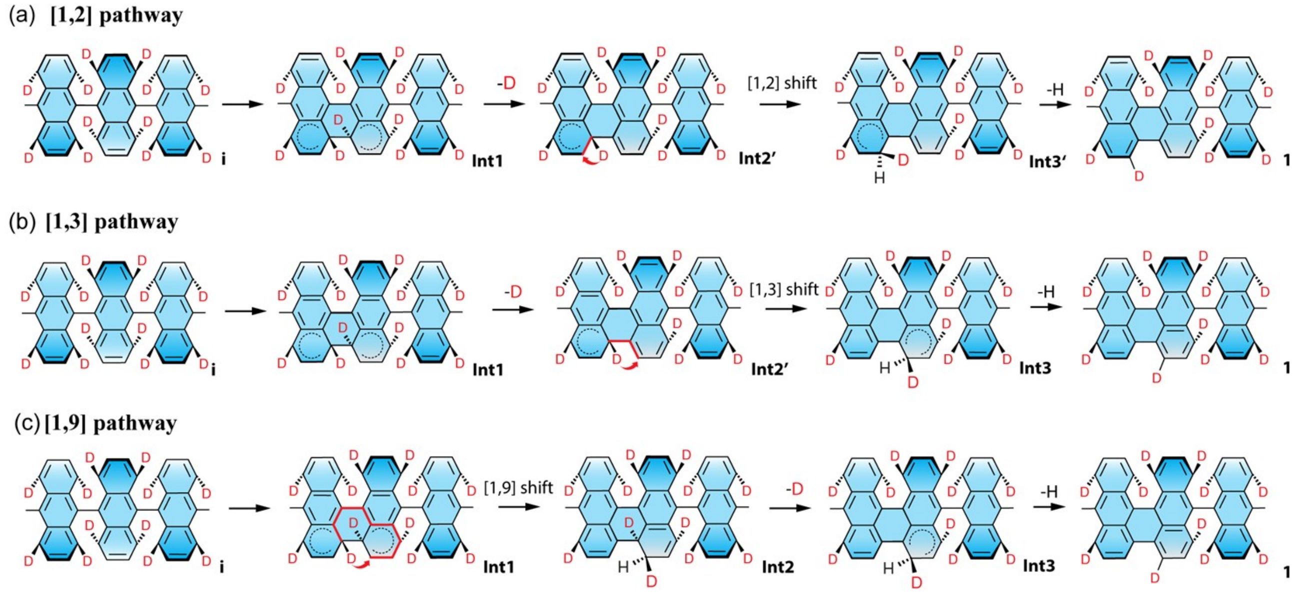


**Fig. S15** CI-NEB simulated pathways, corresponding to the energy profiles in Fig. 4b in the main text, in relation to the formation of the first C–C bond. (a) The [1,2] pathway, which follows the [1,2] hydrogen shift as proposed by Blankenburg et al.,[18] but modified by directly desorbing the edge hydrogen facing down to the Au surface after the formation of one C–C bond. (b) The [1,3] pathway considering a suprafacial [1,3]-sigmatropic hydrogen shift, as shown in Fig. S1$d_1$. (c) The [1,9] pathway, same as Fig. 4a in the main text, while only considering one C–C bond formation. The three pathways share the same initial (state **i**), **Int1**, and final (state **f**) states. The [1,2] and [1,3] pathways have the same **Int2’**, which is different from the **Int2** in the [1,9] pathway, due to different H elimination orders. The [1,9] and [1,3] pathways have the same **Int3**, which is different from the **Int3’** in the [1,2] pathway, due to the different H shifts. One can see that the [1,3] pathway involves a hydrogen shift across the fjord of bond formation, similar to that in the [1,9] pathway.

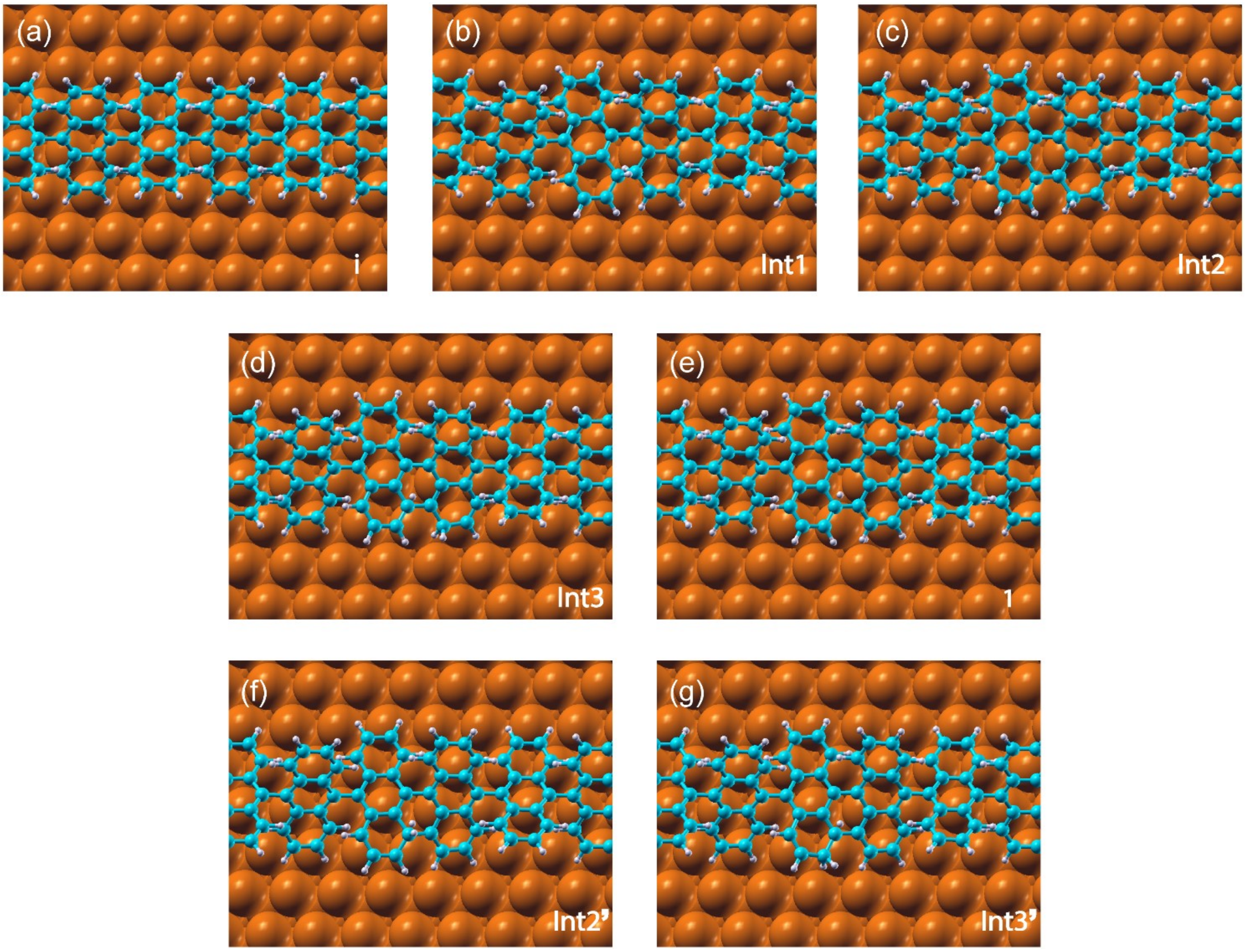


**Fig. S16** Top views for intermediate states on Au(111) surface. (a) initial **i** state, (b) **Int1**, (c) **Int2**, (d) **Int3**, (e) **1** (f) **Int2'**, (g) **Int3'** The periodic directions of the structural models are along the [110] direction of the Au(111).

**Table S1** Calculated energies of intermediate and transition states of the reaction pathways in Fig. S15, with respect to the energy of the initial state **i**.

| [1,2] pathway | | [1,3] pathway | | [1,9] pathway | |
|---|---|---|---|---|---|
| Species | *E* (eV) | Species | *E* (eV) | Species | *E* (eV) |
| **i** | 0.00 | **i** | 0.00 | **i** | 0.00 |
| **Trs1** | 2.22 | **Trs1** | 2.22 | **Trs1** | 2.22 |
| **Int1** | 2.16 | **Int1** | 2.16 | **Int1** | 2.16 |
| **Trs2’** | 3.30 | **Trs2’** | 3.30 | **Trs2** | 3.13 |
| **Int2’** | 2.20 | **Int2’** | 2.20 | **Int2** | 0.44 |
| **Trs3”** | 3.23 | **Trs3’** | 2.95 | **Trs3** | 1.70 |
| **Int3’** | 1.27 | **Int3** | 1.26 | **Int3** | 1.26 |
| **Trs4’** | 2.24 | **Trs4** | 2.02 | **Trs4** | 2.02 |
| **1** | 1.73 | **1** | 1.73 | **1** | 1.73 |

## References


1. X. Liang and S. Duttwyler, *Asian J. Org. Chem.*, 2017, **6**, 1063–1071.
2. C. Ma, L. Liang, Z. Xiao, A. A. Puretzky, K. Hong, W. Lu, V. Meunier, J. Bernholc and A.-P. Li, *Nano Lett.*, 2017, **17**, 6241–6247.
3. C. Ma, Z. Xiao, A. A. Puretzky, A. P. Baddorf, W. Lu, K. Hong, J. Bernholc and A.-P. Li, *Phys. Rev. Materials*, 2018, **2**, 014006.
4. G. Kresse and J. Furthmüller, *Comput. Mater. Sci.*, 1996, **6**, 15–50.
5. A. Togo and I. Tanaka, *Scr. Mater.*, 2015, **108**, 1–5.
6. L. Liang and V. Meunier, *Nanoscale*, 2014, **6**, 5394–5401.
7. L. Talirz, H. Söde, T. Dumslaff, S. Wang, J. R. Sanchez-Valencia, J. Liu, P. Shinde, C. A. Pignedoli, L. Liang, V. Meunier, N. C. Plumb, M. Shi, X. Feng, A. Narita, K. Müllen, R. Fasel and P. Ruffieux, *ACS Nano*, 2017, **11**, 1380–1388.
8. R. Denk, M. Hohage, P. Zeppenfeld, J. Cai, C. A. Pignedoli, H. Söde, R. Fasel, X. Feng, K. Müllen, S. Wang, D. Prezzi, A. Ferretti, A. Ruini, E. Molinari and P. Ruffieux, *Nat. Commun.*, 2014, **5**, 4253.
9. M. Di Giovannantonio, O. Deniz, J. I. Urgel, R. Widmer, T. Dienel, S. Stolz, C. Sanchez-Sanchez, M. Muntwiler, T. Dumslaff, R. Berger, A. Narita, X. Feng, K. Mullen, P. Ruffieux and R. Fasel, *ACS Nano*, 2018, **12**, 74–81.
10. C. Bronner, J. Björk and P. Tegeder, *J. Phys. Chem. C*, 2015, **119**, 486–493.
11. P. Giannozzi, S. Baroni, N. Bonini, M. Calandra, R. Car, C. Cavazzoni, D. Ceresoli, G. L. Chiarotti, M. Cococcioni, I. Dabo, A. D. Corso, S. de Gironcoli, S. Fabris, G. Fratesi, R. Gebauer, U. Gerstmann, C. Gougoussis, A. Kokalj, M. Lazzeri, L. Martin-Samos, N. Marzari, F. Mauri, R. Mazzarello, S. Paolini, A. Pasquarello, L. Paulatto, C. Sbraccia, S. Scandolo, G. Sclauzero, A. P. Seitsonen, A. Smogunov, P. Umari and R. M. Wentzcovitch, *J. Phys.: Condens. Matter.*, 2009, **21**, 395502.
12. D. Vanderbilt, *Phys. Rev. B*, 1990, **41**, 7892–7895.
13. J. P. Perdew, K. Burke and M. Ernzerhof, *Phys. Rev. Lett.*, 1996, **77**, 3865–3868.
14. T. Thonhauser, V. R. Cooper, S. Li, A. Puzder, P. Hyldgaard and D. C. Langreth, *Phys. Rev. B*, 2007, **76**, 125112.

15. Z. Xiao, C. Ma, W. Lu, J. Huang, L. Liang, K. Hong, A.-P. Li, B. G. Sumpter and J. Bernholc, *Npj Comput. Mater.*, 2019, **5**, 91.
16. G. Henkelman, B. P. Uberuaga and H. Jónsson, *J. Chem. Phys.*, 2000, **113**, 9901–9904.
17. J. Björk, S. Stafstrom and F. Hanke, *J. Am. Chem. Soc.*, 2011, **133**, 14884 – 14887.
18. S. Blankenburg, J. Cai, P. Ruffieux, R. Jaafar, D. Passerone, X. Feng, K. Müllen, R. Fasel and C. A. Pignedoli, *ACS Nano*, 2012, **6**, 2020–2025.
19. Z. Xiao, C. Ma, J. Huang, L. Liang, W. Lu, K. Hong, B. G. Sumpter, A. P. Li and J. Bernholc, *Adv. Theory Simul.*, 2018, **2**, 1800172.
20. C. Ma, Z. Xiao, J. Huang, L. Liang, W. Lu, K. Hong, B. G. Sumpter, J. Bernholc and A.-P. Li, *Phys. Rev. Mater.*, 2019, **3**, 016001.
21. F. R. Lucci, M. T. Darby, M. F. G. Mattera, C. J. Ivimey, A. J. Therrien, A. Michaelides, M. Stamatakis and E. C. H. Sykes, *J. Phys. Chem. Lett.*, 2016, **7**, 480–485.
22. C. Ma, Z. Xiao, H. Zhang, L. Liang, J. Huang, W. Lu, B. G. Sumpter, K. Hong, J. Bernholc and A.-P. Li, *Nat. Commun.*, 2017, **8**, 14815.
23. C. Ma, Z. Xiao, A. A. Puretzky, A. P. Baddorf, W. Lu, K. Hong, J. Bernholc and A.-P. Li, *Phys. Rev. Mater.*, 2018, **2**, 014006.
24. I. A. Verzhbitskiy, M. D. Corato, A. Ruini, E. Molinari, A. Narita, Y. Hu, M. G. Schwab, M. Bruna, D. Yoon, S. Milana, X. Feng, K. Müllen, A. C. Ferrari, C. Casiraghi and D. Prezzi, *Nano Lett.*, 2016, **16**, 3442–3447.
25. A. P. C. Reed, R. M. Lambert and J. S. Foord, *Surf. Sci.*, 1983, **134**, 689–702.
26. U. Bardi and G. Rovida, *Surf. Sci.*, 1983, **128**, 145–168.
27. A. Mairena, M. Baljozovic, M. Kawecki, K. Grenader, M. Wienke, K. Martin, L. Bernard, N. Avarvari, A. Terfort, K.-H. Ernst and C. Wäckerlin, *Chemical Science*, 2019, **10**, 2998-3004.
28. K. Wandelt, ed., *Solid-Gas Interfaces I*, Wiley-VCH, Weinheim, Germany, 2015.
29. C. Wackerlin, *Angew. Chem. Int. Ed.*, 2021, **60**, 8446-8449.
